# Naringenin modulates oxidative stress and lipid metabolism: Insights from network pharmacology, mendelian randomization, and molecular docking

**DOI:** 10.3389/fphar.2024.1448308

**Published:** 2024-10-15

**Authors:** Jian Gao, Linjie Yuan, Huanyu Jiang, Ganggang Li, Yuwei Zhang, Ruijun Zhou, Wenjia Xian, Yutong Zou, Quanyu Du, Xianhua Zhou

**Affiliations:** ^1^ Department of Endocrinology, Hospital of Chengdu University of Traditional Chinese Medicine, Chengdu, Sichuan, China; ^2^ Traditional Chinese Medicine Hospital of Meishan, Chengdu, China; ^3^ School of Basic Medicine, Chengdu University of Traditional Chinese Medicine, Chengdu, Sichuan, China; ^4^ Department of Geriatrics, Hospital of Chengdu University of Traditional Chinese Medicine, Chengdu, Sichuan, China; ^5^ TCM Regulating Metabolic Diseases Key Laboratory of Sichuan Province, Chengdu, Sichuan, China

**Keywords:** naringenin, oxidative stress, lipid metabolism, mendelian randomization, hyperlipidemia, proprotein convertase subtilisin/kexin type 9, apolipoprotein B, neurocan

## Abstract

**Background:**

Previous studies have demonstrated that naringenin possesses lipid-lowering effects; however, the underlying mechanisms, particularly its specific molecular targets, remain uncertain.

**Methods:**

Using bioinformatics, three traditional Chinese medicine databases and one human disease database were integrated to establish two naringenin-target-hyperlipidemia modules: naringenin-oxidative stress (OS) and naringenin-lipid metabolism (LM). Data on 1,850 proteins from 1,871 genetic instruments were sourced from seven previous studies. Using Mendelian randomization based on data from the Integrative Epidemiology Unit genome-wide association study (case, n = 5,153; control, n = 344,069), we identified potential drug targets that were subsequently validated in the UK Biobank (396,565 individuals) and FinnGen (412,181 individuals) cohorts. Using molecular docking and molecular dynamics simulation to verify the binding ability of naringenin and causal protein.

**Results:**

In plasma, every standard deviation increase in apolipoprotein B (APOB) was associated with an increased risk of hyperlipidemia (odds ratio [OR] = 9.37, 95% confidence interval [CI], 5.12–17.12; *P* = 3.58e-13; posterior probability of hypothesis 4 [PPH4] = 0.997), and the same was observed for proprotein convertase subtilisin/kexin type 9 (OR = 1.81, 95% CI, 1.51–2.16; *P* = 6.87e-11; PPH4 = 1) and neurocan (OR = 2.34, 95% CI, 1.82–3.01; *P* = 4.09e-11; PPH4 = 0.932). The intersection of two modules and Mendelian randomization result identified APOB as a key regulatory target of naringenin in the treatment of hyperlipidemia. The binding energy between naringenin and APOB was determined to be −7.7 kcal/mol. Additionally, protein-protein interactions and protein-disease networks were analyzed to uncover potential connections between proteins and hyperlipidemia.

**Conclusion:**

This Mendelian randomization-based combined analysis offers a robust framework for elucidating the pharmacological effects of naringenin and identifying candidate proteins for further investigation in the context of hyperlipidemia treatment.

## 1 Introduction

Hyperlipidemia is a prevalent condition characterized by disrupted lipid metabolism (LM) (Natesan and Kim, 2021). It is defined as elevated levels of total cholesterol, triglycerides, low-density lipoprotein (LDL), and lipoprotein above the 90th percentile or high-density lipoprotein levels below the 10th percentile, relative to the general population (Hill and Bordoni, 2024). Hyperlipidemia is a significant risk factor for numerous health issues including diabetes mellitus, obesity, hypertension, cardiovascular disease, cerebrovascular disease, and fatty liver disease (Karr, 2017). It increases the risk of cardiovascular disease by approximately two-fold. In recent years, lifestyle and food safety concerns have contributed to the increasing global incidence of hyperlipidemia (álvarez et al., 2020; Enani et al., 2020). The identification of effective intervention strategies remains a crucial area of clinical focus.

Lipid-lowering drugs, including statins and fibrates, are important in the management of hyperlipidemia (Last et al., 2011). However, the limitations of these two classes of drugs include treatment resistance, intolerance owing to adverse reactions, and lack of compliance (Wołowiec et al., 2023; German and Shapiro, 2020). Additionally, strict clinical indications such as the recommended LDL-cholesterol level limit the use of these drugs. The treatment of familial hypercholesterolemia is challenging, and in addition to high-dose combined statins or other drugs, lomitapide, mipomersen, or LDL-cholesterol removal is usually required (Versmissen et al., 2008). In combination therapy, natural herbal medicines are an important supplement and adjuvant to lipid-lowering drugs that can promote blood lipid levels to reach their target (Rauf et al., 2022). Simultaneously, natural medicines can also reduce the adverse reactions of lipid-lowering drugs and increase patient tolerance (Mulvihill et al., 2009). Previous studies have shown that naringenin, a flavonoid extracted from the traditional Chinese herbal medicine Qingpi (Latin name Citri Reticulatae Pericarpium Viride), has lipid-lowering and antioxidant properties, and may improve hyperlipidemia through multiple pathways (Raja et al., 2019). These effects have been verified in Traditional Chinese Medicine (TCM) prescriptions and experimental animal models and are new types of lipid-lowering and adjuvant therapies with potential (Jung et al., 2003; Yu et al., 2022). Whether the previously investigated mechanism of action of naringenin is causally linked to hyperlipidemia remains uncertain. Current research often relies solely on network pharmacology, which primarily identifies potential drug targets without confirming their direct causal effects on diseases (Miao et al., 2022). This limitation highlights the need for complementary methods, such as Mendelian randomization (MR), to validate the functional relevance of these targets in disease contexts.

Human plasma proteins are critical components in various biological processes, and serve as important drug targets (Rucevic et al., 2011). Many studies have demonstrated that protein drug targets supported by genetic associations have a significantly increased the likelihood of approval (Nelson et al., 2015). MR analysis has recently gained considerable attention in drug target development (Birney, 2022; Reay and Cairns, 2021). Through genetic instrumental variable analysis using single nucleotide polymorphisms (SNPs) from genome-wide association study (GWAS) summary level data, MR can be used to estimate the causal effect of exposure on the outcome (Birney, 2022; Sanderson et al., 2022). With the advancement of high-throughput proteomic and genomic technologies, integrating GWAS and protein quantitative trait loci (pQTL) data for hyperlipidemia can enhance the accuracy of drug targets and indication selection through MR studies (Chong et al., 2019).

Molecular docking (MD) and molecular dynamics simulations (MDS) are essential tools for predicting the interactions between small molecules and their target proteins, providing insights into binding affinities and the stability of these complexes (Alonso et al., 2006). In our study, MD and MDS were employed to investigate the interactions between naringenin and its target proteins at the molecular level. These methods provide detailed insights into the binding affinities and stability of naringenin-protein complexes, which are essential for understanding the potential effects of naringenin on hyperlipidemia. By integrating these methods with MR analysis, we not only identified potential drug targets but also confirmed their direct causal impact on disease outcomes.

**FIGURE 1:**
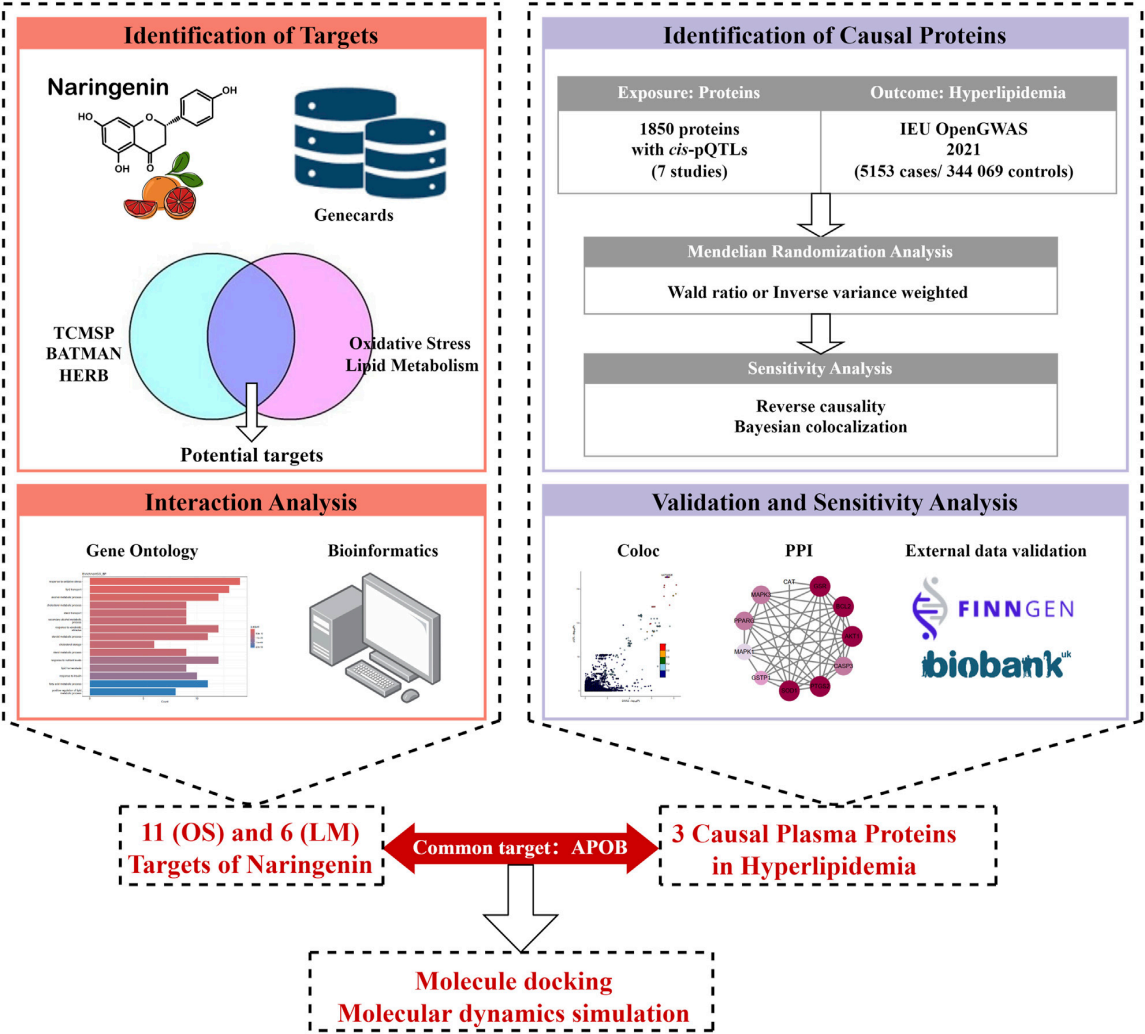
Study design for identification of naringenin targets and causally associated of those targets with hyperlipidemia.

The study design is illustrated in Figure 1. First, we integrated three TCM databases to screen naringenin targets and provide their biological interpretations (Ru et al., 2014; Kong et al., 2024; Fang et al., 2021). Second, we identified potential causal proteins of hyperlipidemia using MR from the Integrative Epidemiology Unit (IEU) OpenGWAS data and summarized seven pQTL datasets (Sun et al., 2018; Ferkingstad et al., 2021; Pietzner et al., 2021; Sun et al., 2023; Suhre et al., 2017; Yao et al., 2018; Folkersen et al., 2017). Third, we performed sensitivity analyses using the Bayesian co-localization and reversed causality detection for preliminary validation and further screening. Fourth, the above results were explained by protein-protein interaction (PPI) and protein-protein MR analyses. Fifth, we performed external validation using datasets from the UK Biobank and FinnGen. Finally, MD and MDS were employed to evaluate the binding ability of naringenin with the causal protein.

## 2 Materials and methods

### 2.1 Naringenin potential targets for hyperlipidemia

Naringenin targets were obtained from the Traditional Chinese Medicine Systems Pharmacology Database and Analysis Platform (version 2.3; https://old.tcmsp-e.com/tcmsp.php) (Ru et al., 2014), HERB (http://herb.ac.cn/) (Fang et al., 2021), and Bioinformatics Annotation Database for Molecular Mechanism of Traditional Chinese Medicine (version 2.0; http://bionet.ncpsb.org.cn/batman-tcm) (Kong et al., 2024). The required oral bioavailability was >0.3 and drug-likeness was >0.18 (Jin et al., 2022). Drug-target networks of the three databases were then summarized and duplicates were removed. In the Human Gene Database (GeneCards), all the protein targets were searched for “oxidative stress” and “lipid metabolism” to establish a disease-target network and remove duplicates (Stelzer et al., 2016). Biological function analysis of naringenin targets was performed using the Gene Ontology (GO, https://geneontology.org/) database, and the results were plotted using the R version 4.4.0 “clusterProfiler” package. Subsequently, the naringenin-oxidative stress (OS) module for naringenin and OS and naringenin-LM module for naringenin and LM were created by taking the intersection of the drug targets and disease targets.

### 2.2 Plasma pQTL

We obtained plasma pQTLs from summary data of seven previously published GWAS studies (Sun et al., 2018; Ferkingstad et al., 2021; Pietzner et al., 2021; Sun et al., 2023; Suhre et al., 2017; Yao et al., 2018; Folkersen et al., 2017) and set pQTL inclusion criteria as follows: (i) exhibited genome-wide significant associations (*P* < 5e−08); (ii) located outside the major histocompatibility complex region (chr6, 26–34 Mb); (iii) demonstrated independence (linkage disequilibrium clumping *r* ^2^ < 0.001); and (iv) *cis*-acting pQTLs (Lin et al., 2023). Ultimately, 1,871 *cis*-pQTLs for 1,850 proteins were identified.

Data from two previously published plasma pQTL studies were used for external validation. Additionally, we referenced the corresponding Genome Reference Consortium Human Build 38 (Nurk et al., 2022) to complete the QTL GWAS data (Supplementary Table S1).

### 2.3 GWAS summary statistics of hyperlipidemia

The IEU OpenGWAS summary statistics (ebi-a-GCST90104007) were used as primary data source, providing information on 349,222 participants of European ethnicity, including 5,153 cases and 344,069 controls (Trinder et al., 2022). Validation datasets from external sources, including the UK Biobank (up to 2017) and FinnGen study’s R10 release, which included 396,565 (Sudlow et al., 2015) and 412,181 participants, respectively were obtained (Kurki et al., 2023).

### 2.4 Statistical analysis

#### 2.4.1 MR analysis

We used plasma proteins as the exposure and hyperlipidemia as the outcome to perform MR analysis using the R package “TwoSampleMR” (https://github.com/MRCIEU/TwoSampleMR). Genetic instruments were used to test the increased risk of hyperlipidemia per standard deviation increase in plasma protein levels, with a single instrument using the Wald ratio and multiple instruments using the inverse variance-weighted method, followed by heterogeneity analysis.

Preliminary MR multiplex tests were performed using Bonferroni corrections, and proteins prioritized after a threshold *P*-value of 5e-08 (*P* < 5 × 10^−8^) were subjected to further analyses. The threshold for external validation was a *P*-value of 5e-02 (*P* < 5 × 10^−2^). To validate the results, we executed the same variation strategy using similar SNPs employed by genetic instruments in the preliminary analyses.

#### 2.4.2 Reverse causality detection

Following the inclusion criteria for pQTL in the preliminary analysis, 128 hyperlipidemic genetic instruments were identified from the IEU OpenGWAS for bidirectional MR analysis (Supplementary Tables S2 and S3). Data from seven previous studies were used to obtain comprehensive summary statistics for proteins. Estimates were calculated using five statistical methods, including (weighted mode, inverse-variance weighted, weighted median, Egger regression, and simple mode. Steiger filtering was used to determine the orientation of proteins and hyperlipidemia (Hemani et al., 2017). Statistical significance was set at *P* < 0.05.

#### 2.4.3 Bayesian co-localization analysis

We performed Bayesian co-localization analyses using the R package “coloc” (https://github.com/chr1swallace/coloc) to assess the posterior probability that proteins and hyperlipidemia share similar SNPs. This method was used to assess the posteriori probabilities for five hypotheses, as previously mentioned (Lin et al., 2023), regarding whether a single variant was shared between the two features. In this study, we focused on the posterior probability of hypothesis 4 (PPH4), which suggests that both proteins and hyperlipidemia are associated with this region through covariation. We used coloc. abf to define >90% of SNP-based PPH4 as evidence of co-localization (Burgess et al., 2019).

### 2.5 PPI and protein-hyperlipidemia association

All PPI and protein-associated disease analyses were performed using Search Tool for Retrieving Interacting Genes database, version 11.5 (Szklarczyk et al., 2019), with a minimum interaction score of 0.7, minimum intensity of 0.5, and false discovery rate of <0.05. Causal relationships between proteins analyzed using the Wald ratio method was analyzed using the Bayesian co-localization algorithm and reviewed manually. We concluded that a *P*-value of <0.05 indicates a potential causal role, while a PPH4 score of >0.9 indicates a potentially strong co-localization relationship.

### 2.6 MD analysis of naringenin and APOB

We used semi-flexible docking method to form a stable complex. Naringenin (PubChem CID: 439246) was molecularly docked with protein APOB (Uniprot ID: A0A669KB70) using AutoDock Vina 1.1.2 software (Trott and Olson, 2010). Protein pre-processing, which involved deleting water molecules and redundant ligands and adding hydrogen atoms, was performed using PyMol 2.4 (https://www.pymol.org/). AutoDock Tools 1.5.6 was utilized to generate PDBQT files for docking simulations. The docking box for the protein APOB was set with dimensions of 60 Å × 42 Å × 51 Å and a grid spacing of 1.00 Å. The coordinates of the docking box were set to x: −1.115, y: 5.462, and z: −1.009. All other parameters were kept at their default values. The docking results were configured to output the nine best docking positions. The docking conformation with the lowest binding energy and the highest clustering frequency was considered the most likely binding mode between the ligand and the protein. Finally, PyMol 2.4 was used to visualize the docking results.

### 2.7 MDS analysis of naringenin and APOB

MDS were conducted to evaluate the significance of naringenin in the molecular docking results using GROMACS (version 2021.2). The force fields AMBER99SB-ILDN and AMBER 14SB were applied to naringenin and APOB, respectively. The system was placed in a dodecahedral box filled with TIP3 water molecules and neutralized with NaCl counterions. Periodic boundary conditions were applied. Energy minimization was performed using the steepest descent algorithm with a cutoff of 1.4 nm for Coulomb and van der Waals interactions. The system was equilibrated at 310 K for 100 ps under NVT conditions using a V-rescale thermostat, followed by 100 ps under NPT conditions at 1.0 bar with a Parrinello Rahman barostat, allowing movement of solvent and ions while restraining protein backbones (Bussi et al., 2007). The LINCS algorithm was used for bond constraints, and the particle mesh Ewald method managed long-range electrostatics. Following equilibration, the system was simulated for 100 ns at 310 K and 1.0 bar, with trajectory snapshots recorded every 10 ns.

### 2.8 Data availability

The following sources granted access to genome-wide summary-level statistics: the primary study, IEU OpenGWAS, and the UK Biobank. The IEU OpenGWAS summary statistics can be found at https://gwas.mrcieu.ac.uk/, and the UK Biobank GWAS summary statistics data can be downloaded from https://www.leelabsg.org/. The FinnGen (R10 release) dataset was downloaded from https://www.finngen.fi/en/.

## 3 Results

### 3.1 Potential drug targets with naringenin for hyperlipidemia

**FIGURE 2:**
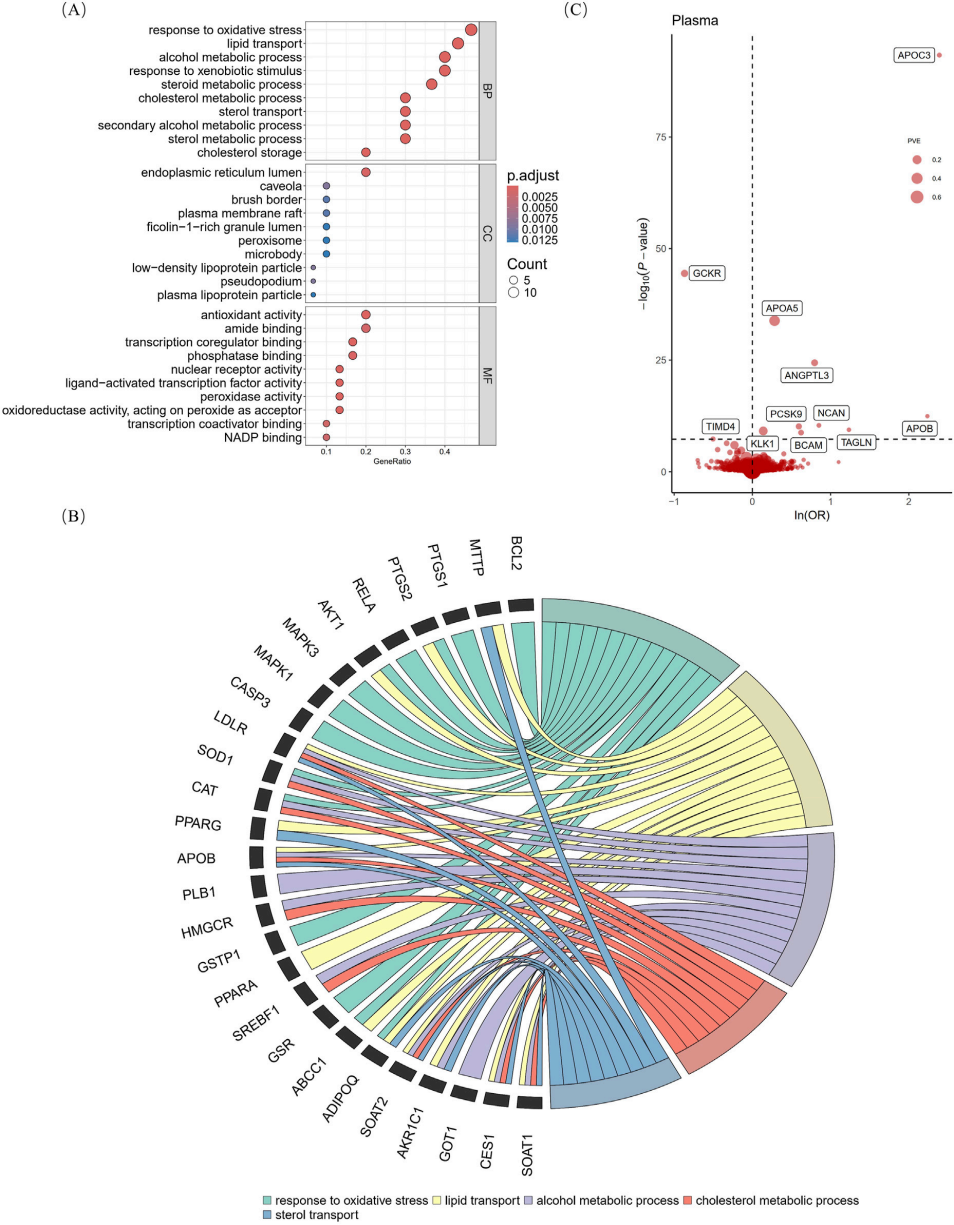
Gene ontology analysis of naringenin targets and primary MR analysis. The vertical axis of Figure **(A)** shows the top 10 enriched terms, and the horizontal axis shows the gene ratio **(B)** shows the correspondence between the top five BP terms sorted by the *p*-value and naringenin targets. **(C)** shows MR analysis with the Wald ratio or inverse variance weighted method of plasma proteins on the risk of hyperlipidemia. The OR for increased risk of hyperlipidemia was expressed as a 10-fold increase in plasma protein levels. The dashed horizontal black line corresponds to *P* = 5e-08. MR, Mendelian randomization; BP, biological process; MF, molecular function; CC, cellular component; p. adjust, *p*-values multiplied by the number of comparisons; ln, natural logarithm; OR, odds ratio; PVE, proportion of variance explained.

The results of searching for “naringenin, target” using three TCM databases showed that after deduplication, there were 30 targets with oral bioavailability of >0.3 and drug similarity of >0.18 (Supplementary Table S4). Biological function analysis of the targets was performed using GO analysis, and the enriched molecular function, cellular component, and biological process results revealed a multitarget mechanism of naringenin. By comparing the adjusted *P* values, we found that the molecular functions of naringenin were mainly antioxidant activity, transcription co-regulator binding, naringenin response to OS, lipid transport, cholesterol metabolism, and in the endoplasmic reticulum lumen (Figure 2A). The top five biological processes and targets were enlarged (Figure 2B) and combined with previous studies, and we proposed that the therapeutic effects of naringenin are mainly achieved through the regulation of OS and LM. By comparing 30 drug targets in the GeneCards database with 131 “oxidative stress” and 38 “lipid metabolism” disease targets, the naringenin-OS module was established, which covers the intersection of targets with an “oxidative stress” relevance score of >20 in the GeneCards database and naringenin targets (Supplementary Table S5). Similarly, the naringenin-LM module comprises the intersection of naringenin with targets that have a “lipid metabolism” relevance score of >10 in GeneCards (Supplementary Table S6), as shown in Supplementary Table S4 and Supplementary Figure S3.

### 3.2 Screening hyperlipidemia-causing proteins from the proteome

According to the Bonferroni significance test (*P* < 5e-08), MR analysis demonstrated a causal relationship between 11 proteins and hyperlipidemia (Table 1; Figure 2C), including angiopoietin-related protein 3 (ANGPTL3), apolipoprotein A-V (APOA5), apolipoprotein C-III (APOC3), apolipoprotein B (APOB)-100, basal cell adhesion molecule (BCAM), glucokinase regulatory protein (GCKR), kallikrein-1 (KLK1), neurocan core protein (NCAN), protein convertase subtilisin/kexin type 9 (PCSK9), transgelin (TAGLN), and T-cell immunoglobulin and mucin domain-containing protein 4 (TIMD4). Specifically, increased levels of GCKR (odds ratio [OR] = 0.42, 95% confidence interval [CI], 0.37–0.47; *P* = 3.64e-45) and TIMD4 (OR = 0.60, 95% CI, 0.50–0.72; *P* = 4.43e-08) decreased the risk of hyperlipidemia. In contrast, incensed levels of ANGPTL3 (OR = 2.21, 95% CI, 1.91–2.57; *P* = 3.88e-25), APOA5 (OR = 1.33, 95% CI, 1.27–1.39; *P* = 1.45e-34), APOB (OR = 9.37, 95% CI, 5.12–17.12; *P* = 3.58e-13), APOC3 (OR = 10.91, 95% CI, 8.69–13.70; *P* = 4.41e-94), BCAM (OR = 1.86, 95% CI, 1.52–2.28; *P* = 1.65e-09), KLK1 (OR = 1.15, 95% CI, 1.10–1.20; *P* = 7.15e-10), NCAN (OR = 2.34, 95% CI, 1.82–3.01; *P* = 4.09e-11), TAGLN (OR = 3.44, 95% CI, 2.33–5.05; *P* = 3.78e-10), and PCSK9 (OR = 1.81, 95% CI, 1.51–2.16; *P* = 6.87e-11) indicated higher risk of hyperlipidemia.

**TABLE 1:** Preliminary Mendelian randomization results for plasma proteins significantly associated with hyperlipidemia after Bonferroni correction.

Protein	UniProt ID	SNP	Effect allele	Or (95% CI)	*p*-Value	PVE (%)	F Statistics
ANGPTL3	Q9Y5C1	rs11207970	T	2.21 (1.91, 2.57)	3.88E-25	6.82	401.92
APOA5	Q6Q788	rs3135506	C	1.33 (1.27, 1.39)	1.45E-34	34.14	3557.63
APOB	P04114	rs563290	G	9.37 (5.12, 17.12)	3.58E-13	0.23	69.57
APOC3	P02656	rs964184	C	10.91 (8.69, 13.70)	4.41E-94	1.02	107.87
BCAM	P50895	rs28399654	A	1.86 (1.52, 2.28)	1.65E-09	2.88	994.76
GCKR	Q14397	rs1260326	T	0.42 (0.37, 0.47)	3.64E-45	8.73	560.73
KLK1	P06870	rs601338	A	1.15 (1.10, 1.20)	7.15E-10	18.21	22968.89
NCAN	O14594	rs2228603	T	2.34 (1.82, 3.01)	4.09E-11	0.76	538.71
PCSK9	Q8NBP7	rs11591147	T	1.81 (1.51, 2.16)	6.87E-11	3.90	1679.87
TAGLN	Q01995	rs1871757	A	3.44 (2.33, 5.05)	3.78E-10	0.39	52.1
TIMD4	Q96H15	rs4704826	A	0.60 (0.50, 0.72)	4.43E-08	1.47	898.42

PVE, proportion of variance explained; SNPs, single nucleotide polymorphisms.

All SNPs, used were *cis*-acting SNPs.

Odds ratios per standard deviation increased in protein levels as hyperlipidemia risk increased. x.

### 3.3 Sensitivity analysis for hyperlipidemia causal proteins

**FIGURE 3:**
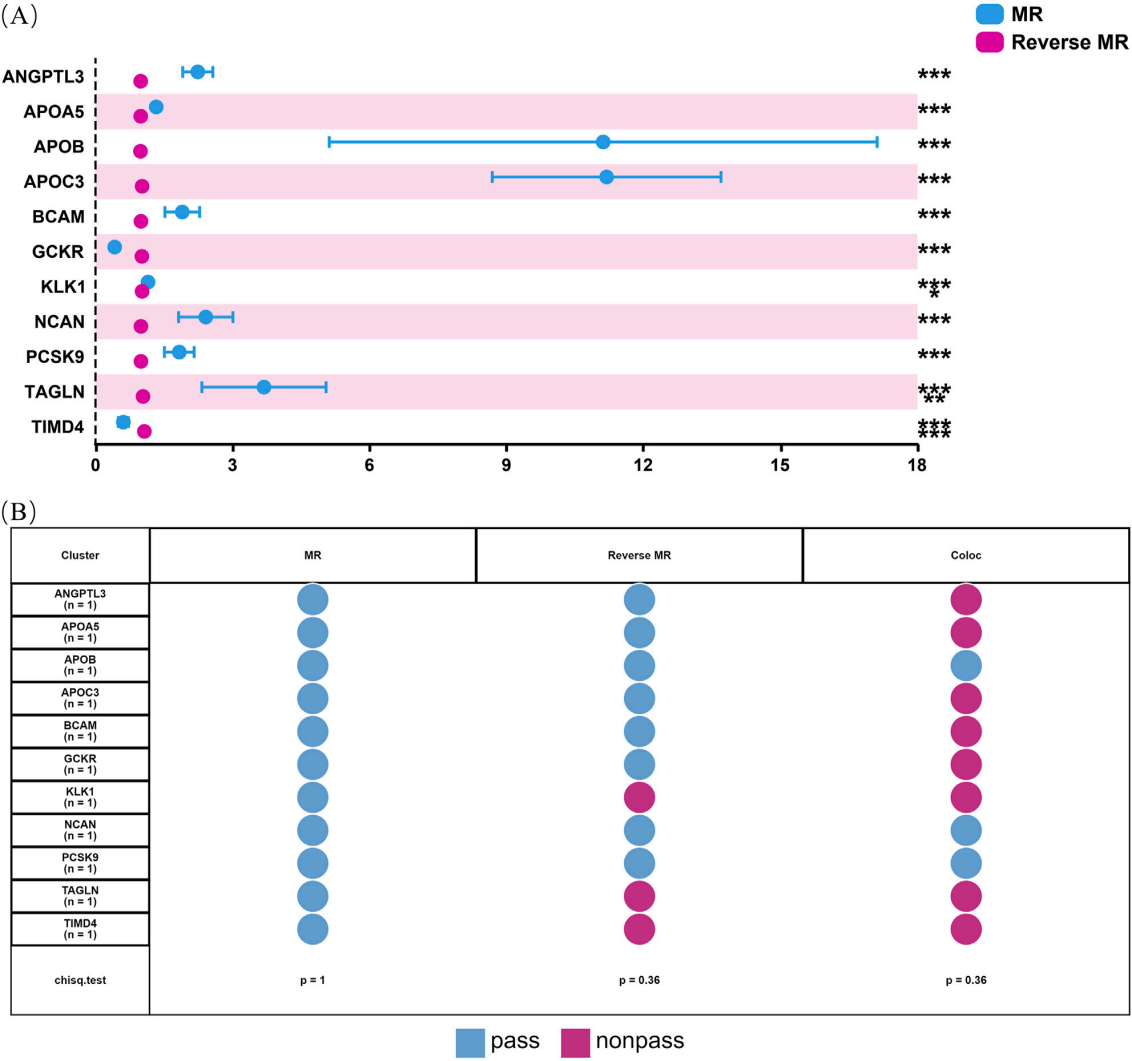
Bidirectional MR analysis of hyperlipidemia on the levels of 11 potential causal proteins. Figure **(A)** shows the results of MR and reverse MR analyses to assess the bidirectional causal relationships between plasma levels of 11 proteins and hyperlipidemia. Blue dots represent MR estimates, indicating the effect of protein levels on hyperlipidemia risk, while pink dots represent Reverse MR estimates, indicating the effect of hyperlipidemia on protein levels. OR are presented per standard deviation increase in plasma protein levels. Error bars represent 95% confidence intervals. Asterisks indicate statistical significance: *p < 0.05, **p < 0.01, ***p < 0.001. **(B)** shows the status of 11 proteins through sensitivity analysis.

Our initial findings revealed that of the eleven causal proteins, nine possessed the potential to serve as drug targets for treating hyperlipidemia. Following the screening of the preliminary analysis results for bidirectional causality, any MR analysis meeting a *p*-value of <0.05 was considered to have reverse causality (Figure 3A). Four potential therapeutic drug targets were identified: APOB, PCSK9, BCAM, and NCAN. Steiger filtering ensures directionality, as listed in Table 2. Bayesian colocalization of three of the four proteins indicated a common variant for hyperlipidemia (Supplementary Figure S1), specifically, APOB (PPH4) = 0.997, NCAN (PPH4 = 0.932), and PCSK9 ([PPH4 = 1], Figure 3B). Additionally, we performed a co-localization analysis of each protein, as shown in Supplementary Figure S2. Notably, PCSK9 shares the same variant as APOB (rs11541192).

**TABLE 2:** Summary of reverse causality detection, Bayesian co-localization analysis and on three potential causal proteins.

Protein	UniprotID	SNP	Bidirectional (MR-IVW)	Steiger filtering	Co-localization PPH4
APOB	P04114	rs1065853	0.97 (0.95, 1.00)	Passed	0.997
NCAN	O14594	rs2228603	0.99 (0.97, 1.00)	Passed	0.932
PCSK9	Q8NBP7	rs11591147	0.99 (0.97, 1.01)	Passed	1

MR-IVW, Mendelian randomization inverse variance-weighted method.

### 3.4 Potential drug targets interaction

PPI network generation using the Search Tool for Retrieving Interacting Genes database (interaction score threshold of 0.7) revealed a strong interaction score between APOB and PCSK9. The results revealed an interaction score of 0.987 for APOB with PCSK9, and the presence of a proven interaction (automated_textmining = 0.985). Interestingly, both APOB and PCSK9 showed strong physical interactions with LDL receptor (LDLR), suggesting that these three proteins are in close proximity to one another, although they may not be in direct contact (Supplementary Figure S4 and Supplementary Table S7). We also established diseases associated with these three proteins using target-disease (strength >0.5; false discovery rate <0.05) analyses, which showed a significant correlation with LM disorders. We hypothesized that APOB and PCSK9 affect lipid levels via multiple pathways (Supplementary Table S8). Additionally, as shown in Supplementary Table S9, we conducted an MR analysis of PCSK9 and APOB and unexpectedly discovered a unidirectional causal relationship (OR = 1.23; 95% CI, 1.05–1.44; *P* = 8.42e-03).

### 3.5 External validation for hyperlipidemia potential drug targets

Validating the primary results by using the same significant variant strategies across different datasets, we observed that NCAN was associated with hyperlipidemia in two external datasets. Similarly, PCSK9 and APOB were associated with hyperlipidemia in the UK Biobank. For example, using the SNPs reported in seven studies as genetic instrumental variables, the risk of hyperlipidemia was augmented by increased APOB (OR = 3.28; 95% CI, 1.78–6.04; *P* = 1.44e-04), NCAN (OR = 1.41; 95% CI, 1.16–1.71; *P* = 4.73e-04), and PCSK9 (OR = 1.39; 95% CI, 1.25–1.54; *P* = 1.00e-09). Additionally, NCAN showed weakly significant causality for hyperlipidemia in the FinnGen cohort (Supplementary Table S10).

### 3.6 Naringenin and causal proteins validation

**FIGURE 4:**
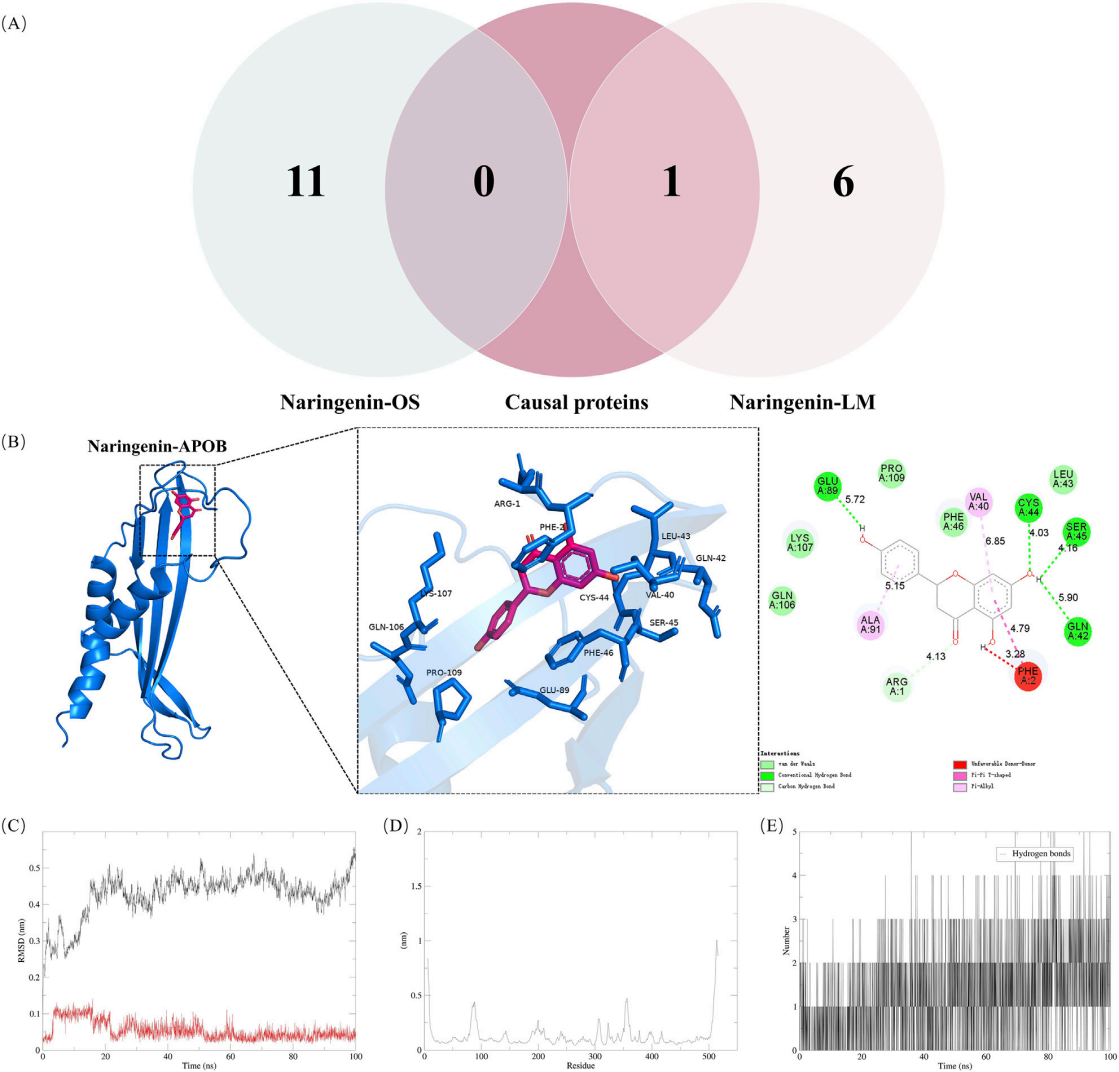
Validation of naringenin targets in treatment of hyperlipidemia **(A)** shows Venn diagram of causal proteins and naringenin-OS and naringenin-LM modules **(B)** shows Docking complexes with the lowest binding energy: naringenin-apolipoprotein **(B)**. Molecular dynamics simulations. The RMSD **(C)** plot, RMSF **(D)** and hydrogen bond numbers **(E)** of naringenin-apolipoprotein **(B)**. RMSD, represents the root mean square deviation; RMSF, root mean square fluctuation.

The Venn diagram analysis of naringenin-related OS and LM using MR identified a common target, APOB, as shown in Figure 4A. MD was employed to explore the optimal binding mode between naringenin and APOB, revealing a binding energy of −7.7 kcal/mol (Supplementary Table S11). An interaction analysis indicated that naringenin formed van der Waals interactions with several nearby amino acids (Figure 4B). The active binding pocket of APOB is composed of amino acid residues including Arg, Cys, Ser, Glu, Phe, Val, Ala, and Gln. The binding pockets of naringenin and APOB exhibit hydrogen bonding and π-π interactions, which stabilize the complex and suggest a high specificity of the interaction. Notably, naringenin also binds to the aromatic amino acid residue Phe, indicating a potential influence on the fluorescence quenching effect of APOB.

To assess the equilibrium time of each naringenin-APOB complex during the MDS, the root mean square deviation (RMSD) of the protein backbone was calculated. RMSD is crucial for estimating molecular conformational changes and determining how long it takes for a system to reach structural equilibrium. Initially, the RMSD values of the simulated complexes (including the reference) typically increase sharply due to the rigidity of the protein in the crystal structure and the restoration of its dynamic motion upon solvation in the water box. As shown in Figure 4C, the x-axis represents time, while the y-axis shows RMSD values. At the beginning of the simulation, the RMSD of the protein-ligand complexes fluctuated significantly. However, after about 20 ns, the RMSD values stabilized, indicating that the systems reached equilibrium. Specifically, naringenin and APOB complexes stabilized around 0.25 nm and 0.05 nm, respectively, suggesting high stability. A higher RMSD indicates a less stable complex; hence, the lower RMSD values for the naringenin-APOB complex reflect its stability under simulated conditions.

To further explore the stability of specific regions within the complexes, root mean square fluctuation (RMSF) analysis was performed. Higher RMSF values indicate regions of higher volatility and less stability. Figure 4D shows that the tail region of naringenin exhibited higher RMSF values, likely due to the presence of tightly coiled structures, such as α-helices and β-sheets. In contrast, lower RMSF values may indicate the loss of corresponding structures within the complex.

Additionally, hydrogen bonds were analyzed to understand their role in naringenin-APOB binding. Hydrogen bonds contribute to binding affinity, with more hydrogen bonds generally indicating stronger binding. As illustrated in Figure 4E, naringenin formed an average of 2 hydrogen bonds with APOB, supporting the stable binding of the complex.

## 4 Discussion

TCM has multi-target effects related to the blood-entry components of its extracts (Zhu et al., 2023; Wu et al., 2022). Therefore, by using multiple databases to summarize the targets and structures of naringenin and setting uniform criteria (oral bioavailability >30%; drug-likeness >0.18), we can more accurately elucidate its mechanism of action (Zhang et al., 2023; Sorokina and Steinbeck, 2020). Through the biological interpretation of GO analyses, we found that naringenin has representative regulatory roles in LM, OS, and other pathways (Figures 2A, B), which is consistent with the results of many previous studies (Zaidun et al., 2018; Ji et al., 2022; Xu et al., 2023). This result was confirmed by establishing target-disease modules using the GeneCards datasets (Supplementary Tables S2 and S3).

An imbalance between oxidants and antioxidants results in OS, leading to the disruption of redox signaling regulation and cellular and molecular damage (Sies, 2015). Lipid peroxidation is a direct effect of OS on LM(Masenga et al., 2023). Reactive oxygen species (ROS), including superoxide and hypochlorous acid, react with polyunsaturated fatty acids (Barrera, 2012). The products of lipid peroxidation decomposition have long half-lives and are readily diffusible (Gentile et al., 2017). The final product of lipid peroxidation, 4-hydroxynonenal, is involved in metabolic regulation, aggravates cell membrane damage, and is considered the secondary messenger of OS(Ayala et al., 2014; Shoeb et al., 2014). Metabolic disorders, such as type 2 diabetes mellitus and obesity, frequently exhibit dysregulated LM, persistent inflammation, and OS in the adipose tissue (Galicia-Garcia et al., 2020). Malondialdehyde, a factor that can affect LM, modified proteins and alters the rate of fatty acid synthesis (Ayala et al., 2014). OS can also affect LM by modulating several signaling pathways such as adenosine 5′-monophosphate (AMP)-activated protein kinase, which is a central regulator of cellular energy metabolism (Fang et al., 2022). However, AMP-activated protein kinase is activated by sustained OS, thereby promoting fatty acid oxidation. Additionally, OS can affect cholesterol and triglyceride metabolism by regulating transcription factors, such as sterol regulatory element-binding protein and peroxisome proliferator-activated receptors (Colak and Pap, 2021). The dysregulation of OS and LM often coexist in metabolic diseases (Manzoor et al., 2022). One of the mechanisms by which lipids accumulate in the vasculature and form emboli is related to ROS over-synthesis, which leads to OS in vascular wall cells and adipocytes. Excessive lipid deposition within vessel walls impedes normal blood flow and causes organ damage (Keeter et al., 2022). Thus, the regulation of OS is one way to intervene in hyperlipidemia.

Naringenin has been reported to treat various OS disorders by improving the activity of superoxide dismutase, catalase, and glutathione and lowering the levels of ROS through various mechanisms (Zaidun et al., 2018; Ji et al., 2022; Xu et al., 2023). Antioxidant activity was validated using various experimental models. However, the underlying mechanisms have not been completely elucidated. To further understand the potential targets of naringenin, we determined that its antioxidant properties were likely attributable to its regulation of OS (Supplementary Table S1). B-cell leukemia/lymphoma 2 protein, alpha serine/threonine-protein kinase, mitogen-activated protein kinase 1, and caspase-3 (Zhang et al., 2019; Huo et al., 2015; Larson-Casey et al., 2016) and the generation of excess ROS in OS mechanisms have been extensively studied. Naringenin inhibited the AMP-activated protein kinase/nicotinamide adenine dinucleotide phosphate oxidase 2/mitogen-activated protein kinase pathway and improved the myocardial hypertrophy caused by OS(Li et al., 2023). Podder et al. showed that naringenin reduced ROS production and upregulated the expression of antioxidant-related genes (Podder et al., 2014). Additionally, naringenin upregulated the antioxidant genes nuclear factor erythroid 2-related factor 2 and heme oxygenase 1, and alleviated OS-induced osteoarthritis (Pan et al., 2022). Several *in vitro* and *in vivo* studies have revealed novel mechanisms for the lipid-lowering effects of naringenin, including modulation of lipid digestion, reversal of cholesterol transport, and LDLR expression (Namkhah et al., 2021; Burke et al., 2018). Our findings (Supplementary Table S2), which are similar to those of previous studies (Mulvihill et al., 2009; Allister et al., 2008), suggest that genetic variants of APOB, microsomal triglyceride transfer protein, LDLR, and 3-hydroxy-3-methylglutaryl-CoA reductase in naringenin-LM are associated with the development of hyperlipidemia (Supplementary Figure S3 and Supplementary Table S3). More interestingly, our study is consistent with a recent study showing that naringenin downregulated the mRNA expression of peroxisome proliferator-activated receptor α in rats (Burke et al., 2018). Among them, naringenin inhibited 3-hydroxy-3-methylglutaryl-CoA reductase and reduced the triglyceride content in adipocytes (Dayarathne et al., 2021). Additionally, it has been demonstrated that OS-induced inflammatory response and high lipid levels can be effectively attenuated by modulating the APOB/sortilin-mediated immune microenvironment (Wu et al., 2024). In summary, we tentatively conclude that the effects of naringenin on OS and LM can be attributed to the regulation of these target proteins.

The “causality” identified by MR may be horizontally pleiotropic or contain reverse causality and genetic confounding. Therefore, proteins with reverse causality were excluded using bidirectional MR, and the results of the Steiger filtering supported our primary findings. We eliminated the bias of horizontal pleiotropy as much as possible using only *cis*-pQTL as an instrumental variable (Chong et al., 2019). Additionally, by applying a Bayesian co-localization threshold of 0.9 for posterior probability, the bias resulting from genetic confounders was successfully mitigated. As shown in Supplementary Figure S1 and Supplementary Table S6, the three proteins identified by co-localization (PCSK9, APOB, and NCAN) may share the same variant. To identify whether naringenin targets are the causal proteins of hyperlipidemia, we integrated results from network pharmacology analyses (naringenin-OS and naringenin-LM modules) with MR findings (Figure 4A).

To the best of our knowledge, this is the first study to reveal a causal relationship between naringenin and hyperlipidemia using a compound-target-disease network and MR. Herein, we report three potential drug-targeting proteins for hyperlipidemia: PCSK9, APOB, and NCAN (Table 2). Among these proteins, the association between APOB and hyperlipidemia was validated using external datasets, which made the results more reliable. Consistent with our findings, previous studies have shown that SNPs in APOB are associated with hyperlipidemia in Chinese and Finnish populations (Lu et al., 2016; Junna et al., 2023). Interestingly, APOB was also a naringenin target (Supplementary Table S4). Consistent with our hypothesis, by establishing a naringenin-target-hyperlipidemia network and inferring a positive causal relationship determined by genetics, we concluded that naringenin can affect LM and treat hyperlipidemia through APOB regulation, and this regulation is likely to have an inhibitory effect. Human APOB is the major protein component of LDL (APOB-100), chylomicron (APOB-48), and very-low-density lipoprotein (APOB-100), and it plays a crucial role in maintaining healthy cholesterol levels. Plasma APOB is equal to the total number of APOB-48 and APOB-100 particles and chylomicrons (Feingold, 2022). Lipid particles that contribute to hyperlipidemia are typically determined by the level of APOB present in blood vessels. Thus, LDL particles with higher cholesterol content are more likely to deposit cholesterol and increase the risk of cardiovascular disease (Behbodikhah et al., 2021). In our study, we observed a significant interaction between APOB and LDLR, which led us to propose that the effects of naringenin may be due to the co-regulation of these two proteins (Supplementary Figure S4 and Supplementary Table S7). Studies have shown that naringenin inhibits APOB secretion in oleic acid-stimulated human hepatocytes and selectively increases rapid APOB degradation (Borradaile et al., 2002).

The results of the combined MR analyses indicate that PCSK9 is a promising therapeutic target (Table 2), as its inhibitor, alirocumab, has been approved by the Food and Drug Administration (Markham, 2015). Additionally, the feasibility of targeting PCSK9 was demonstrated using the newly approved hypercholesterolemic nucleic acid lipid-lowering drug, inclisiran (German and Shapiro, 2020; Wołowiec et al., 2023). According to our results, naringenin did not directly target PCSK9, but it has been reported that it stimulated LDLR (an interacting protein of PCSK9) expression by increasing the phosphorylation of phosphatidylinositol-3 kinase and extracellular signal-regulated protein kinase 1/2, thereby effectively reducing the mortality and morbidity rate of coronary heart disease (Bawazeer et al., 2017). As shown by the results of the MR and PPI analyses between PCSK9 and APOB (Supplementary Tables S7 and S9), PCSK9 and APOB have co-expressed biological patterns and causal relationship. Previous studies have shown that PCSK9 inhibition reduces OS and inflammation in macrophages treated with oxidized LDL (Ding et al., 2018; Cammisotto et al., 2021). Additionally, PCSK9 inhibition can reduce lipid deposition and plaque lesion area and improve vascular OS in patients with high cardiovascular risk (Yang et al., 2023). These findings support our hypothesis that the therapeutic effect of naringenin is reflected in the regulation of the co-expression of proteins related to OS and LM. Although the PPI results showed that NCAN is an isolated node, it is also worth noting that the interaction between NCAN and the environment has been linked to hyperlipidemia, and these studies further support the results of our study (Deng et al., 2020).

MDS indicated that naringenin binds to APOB with a binding affinity of −7.7 kcal/mol (Supplementary Table S11). The stability of this binding was further confirmed through MDS, which highlighted hydrogen bonding as a crucial factor in maintaining the complex’s stability (Figures 4B–E). Our study suggests that naringenin binds stably to APOB, potentially contributing to its therapeutic effects in treating hyperlipidemia.

## 5 Limitations

This study has several limitations. First, there may have been bias arising from the pQTL data sourced from seven different studies. Second, the circulating protein GWAS data were based on aptamers known for their high specificity and stability in binding to target molecules (Song et al., 2022). *Cis*-pQTLs were chosen, and only one SNP was considered, whereas some *trans*-pQTLs were not assessed, which limits the applicability of alternative MR, multiplicity testing, and heterogeneity detection. However, the SNPs utilized in our study were established as strong instrumental variables with F-statistic values > 10, which lends credibility to our statistical analysis (Staiger and Stock, 1997). Third, the data samples employed in our study were derived from European populations, making it challenging to generalize our findings to other populations. Further investigation using more individual data is needed for effective clinical translation of naringenin in the treatment of hyperlipidemia. We also confirmed a causal relationship between APOB, PCSK9, and NCAN and hyperlipidemia in the external dataset (Supplementary Table S10). Further studies among non-European populations are required. Although the PPI findings are encouraging, they should be regarded as suggestive rather than definitive. MD analysis did not capture the interactions with other proteins *in vivo*, but the molecular-level analysis in this study provided a reference and guidance for further exploring the mechanism of naringenin in treating hyperlipidemia.

## 6 Conclusion

This study aimed to investigate whether naringenin exerts therapeutic effects on hyperlipidemia by targeting specific proteins. By integrating network pharmacology, MR analysis, MD, and MDS, our findings suggest that APOB is a key target of naringenin in the treatment of hyperlipidemia. While these results provide valuable insights into the potential mechanisms by which naringenin may influence LM and OS, the exact role of naringenin in modulating APOB remains to be fully elucidated. Therefore, further research is required to clarify the precise molecular pathways involved and to better understand the broader effects of naringenin on LM and OS.

## Data Availability

The datasets presented in this study can be found in online repositories. The names of the repository/repositories and accession number(s) can be found below: https://gwas.mrcieu.ac.uk/, ebi-a-GCST90104007 https://www.leelabsg.org/resources, FinMetSeq https://www.finngen.fi/en/access_results, r10. finngen.fi.
