# Supplementary figures and images for: Naringenin modulates oxidative stress and lipid metabolism: Insights from network pharmacology, mendelian randomization, and molecular docking
Source: Front Pharmacol. 2024 Oct 15;15:1448308. doi: 10.3389/fphar.2024.1448308 (PMC11518751; doi:10.3389/fphar.2024.1448308)

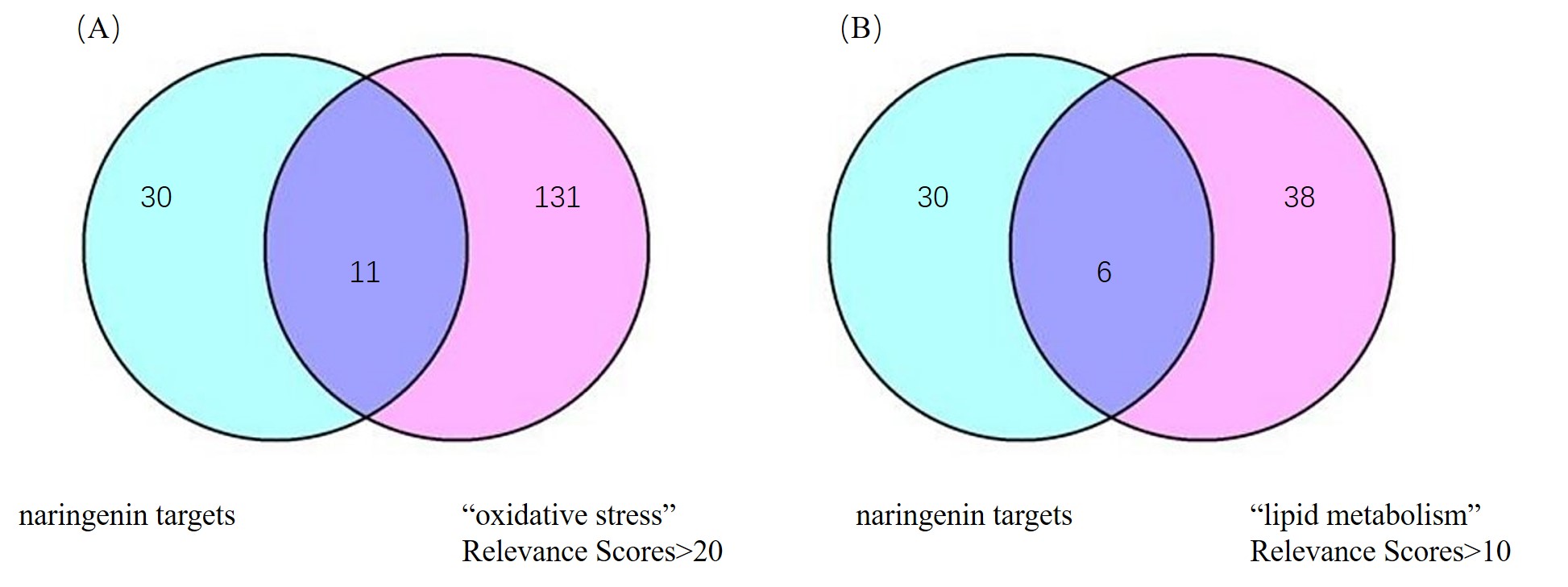

Supplement: Supplementary file 1 [file Image3.JPEG]

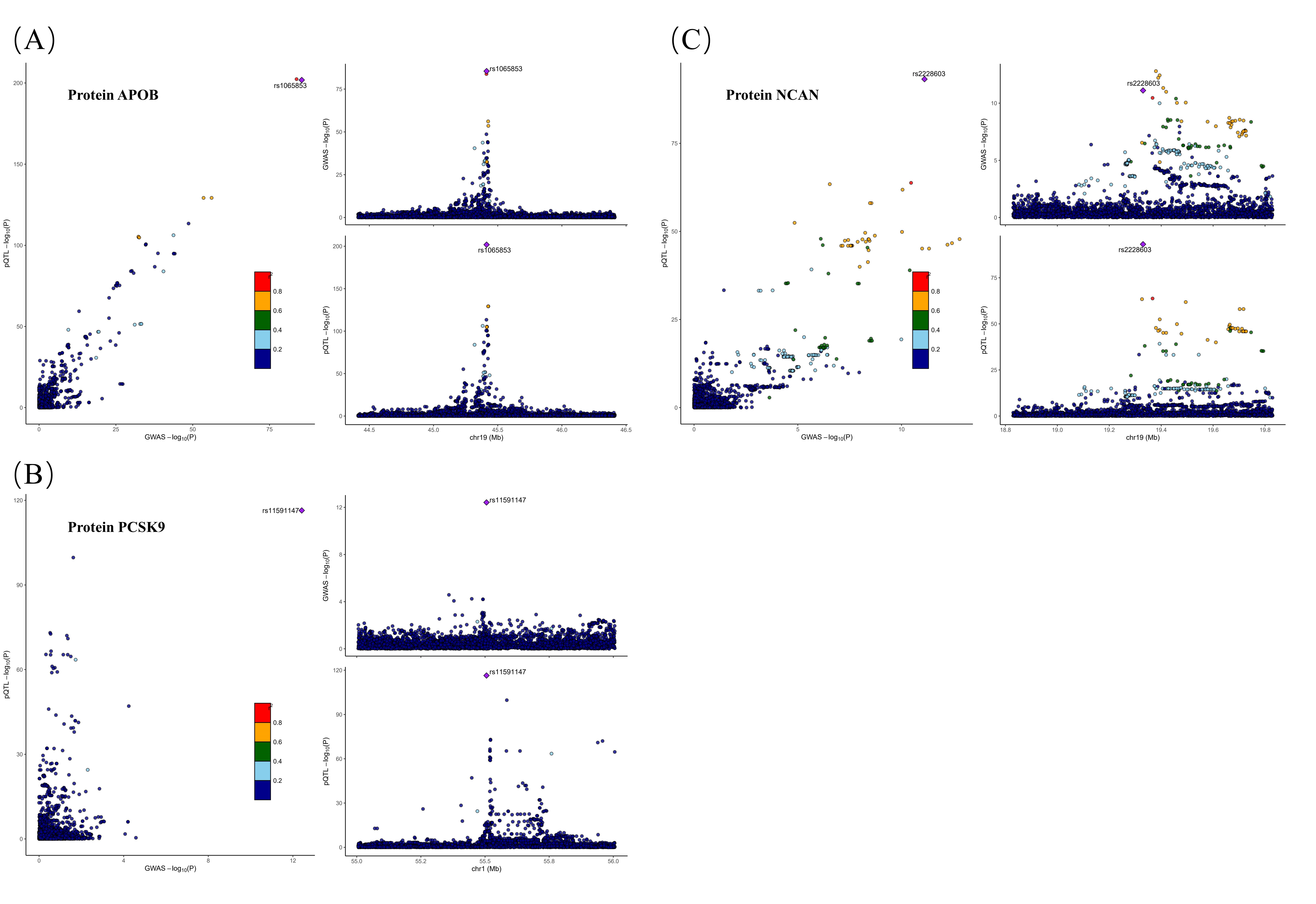

Supplement: Supplementary file 2 [file Image1.JPEG]

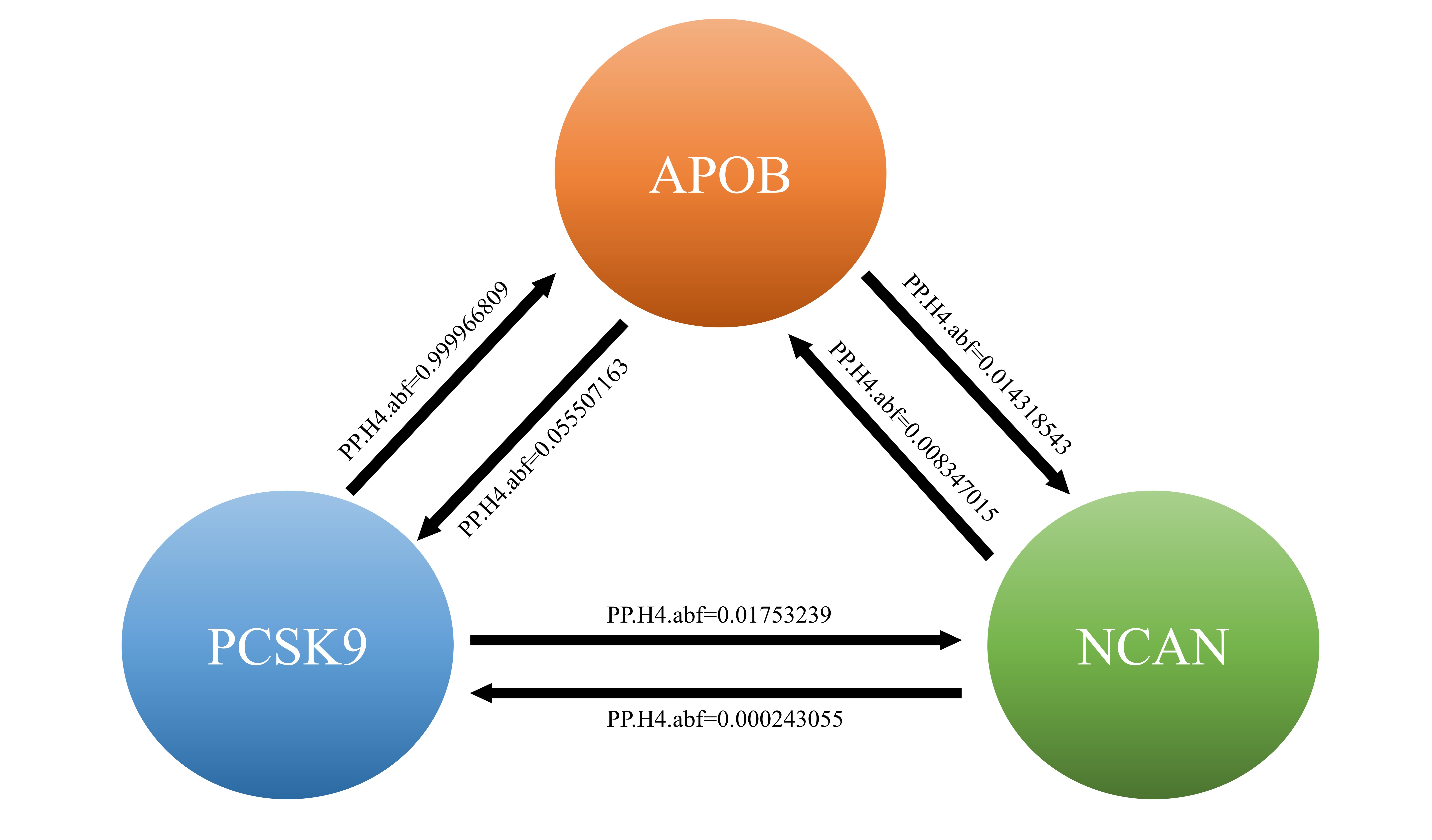

Supplement: Supplementary file 3 [file Image2.JPEG]

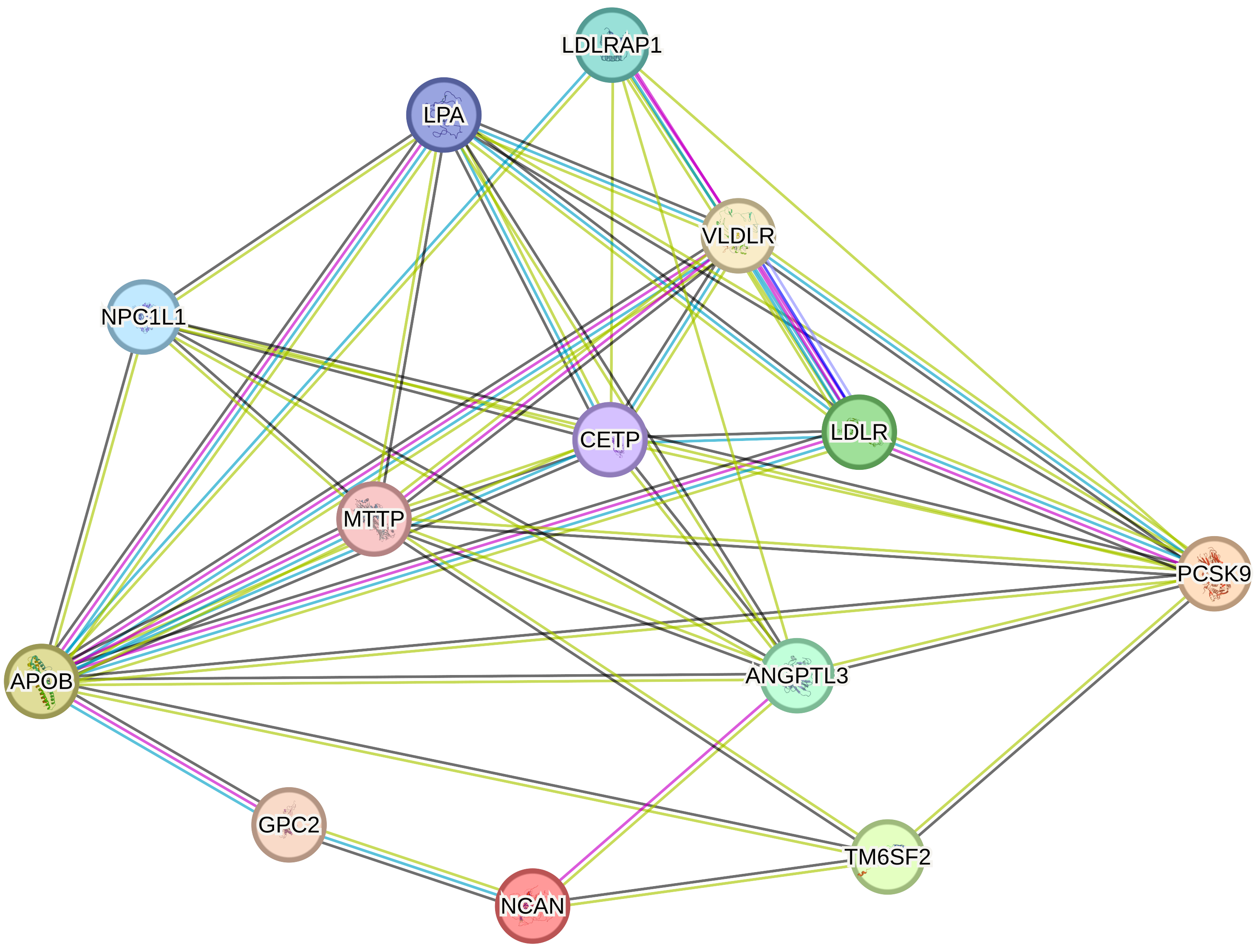

Supplement: Supplementary file 4 [file Image4.PNG]
