# Supplementary material for: Naringenin modulates oxidative stress and lipid metabolism: Insights from network pharmacology, mendelian randomization, and molecular docking
Source: Front Pharmacol. 2024 Oct 15;15:1448308. doi: 10.3389/fphar.2024.1448308 (PMC11518751; doi:10.3389/fphar.2024.1448308)
Supplement: Supplementary file 5 [file Table2.docx]

**STROBE-MR checklist of recommended items to address in reports of Mendelian randomization studies**

| **Item No.** | **Section** | **Description** |  | **Relevant text from manuscript** |
| --- | --- | --- | --- | --- |
| 1 | **TITLE and ABSTRACT** | The study design, data sources, methods, and relevant results of the MR have been described in the title and abstract of this study. |  | **TITLE**  “Naringenin Modulates Oxidative Stress and Lipid Metabolism: Insights from Network Pharmacology, Mendelian Randomization, and Molecular Docking”  **ABSTRACT**  ‘Previous studies have demonstrated that naringenin possesses lipid-lowering effects; however, the underlying mechanisms, particularly its specific molecular targets, remain uncertain. Using bioinformatics, three traditional Chinese medicine databases and one human disease database were integrated to establish two naringenin-target-hyperlipidemia modules: naringenin-oxidative stress (OS) and naringenin-lipid metabolism (LM). Data on 1,850 proteins from 1,871 genetic instruments were sourced from seven previous studies. Using Mendelian randomization based on data from the Integrative Epidemiology Unit genome-wide association study (case, n = 5,153; control, n = 344,069), we identified potential drug targets that were subsequently validated in the UK Biobank (396,565 individuals) and FinnGen (412,181 individuals) cohorts. Additionally, protein-protein interactions and protein-disease networks were analyzed to uncover potential connections between proteins and hyperlipidemia. In plasma, every standard deviation increase in apolipoprotein B (APOB) was associated with an increased risk of hyperlipidemia (odds ratio [OR] = 9.37, 95% confidence interval [CI], 5.12–17.12; P = 3.58e-13; posterior probability of hypothesis 4 [PPH4] = 0.997), and the same was observed for proprotein convertase subtilisin/kexin type 9 (OR = 1.81, 95% CI, 1.51–2.16; P = 6.87e-11; PPH4 = 1) and neurocan (OR = 2.34, 95% CI, 1.82–3.01; P = 4.09e-11; PPH4 = 0.932). The intersection of two modules and Mendelian randomization result identified APOB as a key regulatory target of naringenin in the treatment of hyperlipidemia. The binding energy between naringenin and APOB was determined to be -7.7 Kcal/mol. This Mendelian randomization-based analysis offers a robust framework for elucidating the pharmacological effects of naringenin and identifying candidate proteins for further investigation in the context of hyperlipidemia treatment.’ |
|  | **INTRODUCTION** |  |  |  |
| 2 | **Background** | Hyperlipidemia is a risk factor for many diseases. A large number of experiments have shown that naringenin, an extract of the Chinese herbal medicine Qingpi, is a good drug for resisting oxidative stress and regulating lipid metabolism.  Human plasma proteins play a key role in a series of biological processes and are an important type of drug target. Experiments have shown that protein drug targets supported by genetic associations have a greatly increased chance of obtaining market approval. Recently, MR analysis has been widely used for drug target development and drug repurposing. MR is a genetic instrumental variable analysis that usually uses single nucleotide polymorphisms (SNPs) in genome-wide association studies (GWAS) as genetic tools to estimate the causal effect of exposure on the outcome. Due to the advancement of high-throughput genomic and proteomic technologies in plasma, MR studies integrating GWAS and hyperlipidemia protein quantitative trait loci (pQTL) data can increase the reliability of drug target and indication selection. |  | ‘Current research often relies solely on network pharmacology, which primarily identifies potential drug targets without confirming their direct causal effects on diseases(Miao et al., 2022). This limitation highlights the need for complementary methods, such as Mendelian randomization (MR), to validate the functional relevance of these targets in disease contexts.’  ‘Human plasma proteins are critical components in various biological processes, and serve as important drug targets(Rucevic et al., 2011). Many studies have demonstrated that protein drug targets supported by genetic associations have a significantly increased the likelihood of approval(Nelson et al., 2015). MR analysis has recently gained considerable attention in drug target development(Birney, 2022; Reay and Cairns, 2021). Through genetic instrumental variable analysis using single nucleotide polymorphisms (SNPs) from genome-wide association study (GWAS) summary level data, MR can be used to estimate the causal effect of exposure on the outcome(Birney, 2022; Sanderson et al., 2022). With the advancement of high-throughput proteomic and genomic technologies, integrating GWAS and protein quantitative trait loci (pQTL) data for hyperlipidemia can enhance the accuracy of drug targets and indication selection through MR studies(Chong et al., 2019).’ |
| 3 | **Objectives** | We hypothesized that naringenin could treat hyperlipidemia through its antioxidant and lipid metabolism regulating functions. The aim of this study was to demonstrate by MR analysis whether the target of naringenin (circulating plasma proteins) has a potential causal relationship with hyperlipidemia. |  | ‘The study design is illustrated in Figure 1. First, we integrated three TCM databases to screen naringenin targets and provide their biological interpretations(Ru et al., 2014; Kong et al., 2024; Fang et al., 2021). Second, we identified potential causal proteins of hyperlipidemia using MR from the Integrative Epidemiology Unit (IEU) OpenGWAS data and summarized seven pQTL datasets(Sun et al., 2018; Ferkingstad et al., 2021; Pietzner et al., 2021; Sun et al., 2023; Suhre et al., 2017; Yao et al., 2018; Folkersen et al., 2017). Third, we performed sensitivity analyses using the Bayesian co-localization and reversed causality detection for preliminary validation and further screening. Fourth, the above results were explained by protein-protein interaction (PPI) and protein-protein MR analyses. Fifth, we performed external validation using datasets from the UK Biobank and FinnGen. Finally, MD and MDS were employed to evaluate the binding ability of naringenin with the causal protein.’ |
|  | **METHODS** | **Description** | | |
| 4 | **Study design and data sources** | The primary MR analysis in this study used plasma protein as the exposure and hyperlipidemia as the outcome. The pQTL data were derived from a summary of 7 previously published GWAS studies. Finally, 1871 cis-pQTLs were identified. In addition, two recently published plasma pQTL data were used for external validation. For any missing information in pQTL GWAS summary statistics, such as effect allele frequencies, we used a matched human genome build as a reference to complete the data. Validation datasets from external sources, including the UK Biobank (up to 2017) and the FinnGen study's R10 release were obtained. Following the inclusion criteria for pQTL in the primary analysis, 128 hyperlipidemic genetic instruments were identified from the IEU OpenGWAS for bidirectional MR analysis. Data from seven previous studies were used to obtain comprehensive summary statistics for proteins. Estimates were calculated using five statistical methods include (weighted mode, MR-IVW, weighted median, MR-Egger and simple mode. We used Steiger filtering for orientation determination of proteins and hyperlipidemia. Significance was confirmed by P < 0.05.   \| **Primary MR exposure** \| \| \| \| \| \| --- \| --- \| --- \| --- \| --- \| \| **Exposure** \| **Authors** \| **SNP number** \| **PMID** \| **Sample size** \| \| Plasma protein \| Ferkingstad et al \| 195 \| 34857953 \| 35,559 \| \| Plasma protein \| Folkersen et al \| 19 \| 28369058 \| 30,000 \| \| Plasma protein \| Pietzner et al \| 741 \| 34648354 \| 10,708 \| \| Plasma protein \| Suhre et al \| 66 \| 28397792 \| 1000 \| \| Plasma protein \| Sun_1 et al \| 172 \| 29875488 \| 3301 \| \| Plasma protein \| Sun_2 et al \| 664 \| 37794186 \| 35,571 \| \| Plasma protein \| Yao et al \| 14 \| 30111768 \| 6861 \| \| **Primary MR outcome/BIMR exposure** \| \| \| \| \| \| **Exposure/Outcome** \| **Authors** \| **SNP number** \| **PMID** \| **Sample size** \| \| Hyperlipidemia \| Trinder M et al \| 14,502,301 \| 34906840 \| 349,222 \| \| **BIMR outcome** \| \| \| \| \| \| **Outcome** \| **Authors** \| **SNP number** \| **PMID** \| **Sample size** \| \| 11 plasma proteins \| Ferkingstad et al \| 1,213 \| 34857953 \| 35,559 \| \| **External validation** \| \| \| \| \| \| **Outcome** \| **Study** \| **SNP number** \| **PMID** \| **Sample size** \| \| Hyperlipidaemia \| finn-b-E4_HYPERLIPNAS \| 16,380,389 \| / \| 201,794 \| \| Pure hypercholesterolaemia \| ukb-b-12651 \| 9,851,867 \| / \| 463,010 \| \| Pure hypercholesterolaemia \| finn-b-E4_HYPERCHOL \| 16,380,403 \| / \| 206,067 \| | | |
|  | a) | Setting: The primary MR analysis in this study used plasma protein as the exposure and hyperlipidemia as the outcome. The pQTL data were derived from a summary of 7 previously published GWAS studies. The IEU OpenGWAS summary statistics (ebi-a-GCST90104007) were utilized as the primary outcome data source, providing information on 349,222 participants of European ethnicity, including 5,153 cases and 344,069 controls. Validation datasets from external sources, including the UK Biobank (up to 2017) and the FinnGen study's R10 release (which included 396,565, and 412,181 participants, respectively) were obtained. Following the inclusion criteria for pQTL in the primary analysis, 128 hyperlipidemic genetic instruments were identified from the IEU OpenGWAS for bidirectional MR analysis. Data from seven previous studies were used to obtain comprehensive summary statistics for proteins. Estimates were calculated using five statistical methods include (weighted mode, MR-IVW, weighted median, MR-Egger and simple mode. We used Steiger filtering for orientation determination of proteins and hyperlipidemia. Significance was confirmed by P < 0.05. Research ethics approval was provided in the original study. | | |
|  | b) | Participants: The methods for recruitment, inclusion criteria, and statistical power of the sample size of each study have been reported in the original studies. | | |
|  | c) | pQTL data criteria: (i) showing genome-wide significant association (P < 5e−08); (ii) located outside the major histocompatibility complex (MHC) region (chr6, 26 - 34Mb); (iii) shows independent association [linkage disequilibrium (LD) clustering r^2^<0.001]; and (iv) is a cis-acting pQTL. | | |
|  | d) | Definition: protein quantitative trait loci (pQTLs) are genetic variants that affect the quantity of a specific protein. pQTLs require genome-wide association studies (GWAS) using microarrays, whole-genome sequencing (WGS), or whole-exome sequencing (WES). In our primary MR analysis, cis-pQTLs for human plasma proteins were used as exposures. Familial combined hyperlipidemia defined by Mexico criteria was used as the outcome. In the BIMR analysis, Familial combined hyperlipidemia defined by Mexico criteria was used as the exposure, and pQTLs were used as the outcome. | | |
|  | e) | Research ethics approval was provided in the original study. The sources listed below grant access to genome-wide summary level statistics: the primary study, the IEN OpenGWAS, and the UK Biobank. The IEN OpenGWAS summary statistics can be found at (https://gwas.mrcieu.ac.uk/), while the UK Biobank's GWAS summary statistics can download from (https://www.leelabsg.org/). Additionally, the FinnGen (R10 release) dataset can download at (https://www.finngen.fi/en/). | | |
| 5 | **Assumptions** | The instrumental variables in MR studies must meet three core assumptions: ① there is a robust strong correlation between the instrumental variables and the exposure factors (association hypothesis); ② the instrumental variables are independent of the confounding factors that affect the "exposure-outcome" relationship (independence hypothesis); ③ genetic variation can only affect the outcome through exposure factors and cannot affect the outcome through other pathways (exclusivity hypothesis). | | |
| 6 | **Statistical methods: main analysis** |  | | |
|  | a) | We employed plasma proteins as the exposure and hyperlipidemia as the outcome to perform a MR analysis. | | |
|  | b) | Primary MR multiplex tests were performed using Bonferroni corrections, and proteins prioritized after a threshold of P-value of 5e-08 (P < 5 × 10^-8^) were subjected to further analyses. The threshold for external validation was P-value of 5e-02 (P < 5 × 10^-2^). To validate the primary results, we executed the same variation strategy using the same SNPs as those employed by the genetic instruments in the preliminary analysis. | | |
|  | c) | Genetic instruments were used to test the increased risk of hyperlipidemia per standard deviation (SD) increased in plasma protein levels, with a single instrument using the Wald ratio and multiple instruments using the inverse variance-weighted (MR-IVW) method followed by heterogeneity analysis. | | |
|  | d) | We referenced the corresponding Genome Reference Consortium Human Build 38 (GRCh38) to complete the QTL GWAS data. | | |
| 7 | **Assessment of assumptions** | QTL research has important research significance, and can clarify the quantitative regulatory relationship between DNA sequence variation and quantifiable intermediate molecular phenotypes. At the same time, the data resources generated by these studies are equally important and are widely used in quantitative genetics methods and applications to explain the regulatory association between SNPs and molecules at all levels and the mechanism of the impact of this association on the phenotype. For example, TWAS, COLCO, MAGMA and other method frameworks are developed based on eqtl or pqtl data, and combined with disease GWAS data to map disease-related genes, proteins, etc., thereby revealing the pathogenesis of the disease and screening drug targets. The main issue with the validity of the results of MR analysis is pleiotropy, especially "horizontal pleiotropy", that is, genetic variants do not affect the outcome through the pathway of the risk factor of interest, which will violate the MR assumption and may be caused by multiple biological functions of the gene. Horizontal pleiotropy may lead to spurious non-causal associations between genetic predictors of the risk factor of interest and the outcome, but it may also lead to false negative results if the pleiotropic effects offset the true causal effect of the risk factor on the outcome. The cis-pQTLs we used all had F statistics greater than 10. In addition, the circulating protein GWAS data were all based on aptamers known for their high specificity and stability in binding to target molecules. | | |
| 8 | **Sensitivity analyses and additional analyses** | We used Steiger filtering for orientation determination of proteins and hyperlipidemia, significance was confirmed by P < 0.05. We performed Bayesian co-localization analyses using the "coloc" software to assess the posterior probability that proteins and hyperlipidemia share the same SNPs. This method was used to assess the posteriori probabilities for five hypotheses, as previously mentioned, regarding whether a single variant is shared between two features. In this study, we focused on the posterior probability of hypothesis 4, which suggests that both protein and hyperlipidemia are associated with the region through covariation. We utilized coloc.abf to define greater than 90% of SNP-based PH4 as evidence of co-localization. | | |
| 9 | **Software and pre-registration** |  | | |
|  | a) | Using the R (version 4.4.0) package ‘TwoSampleMR’ (<https://github.com/MRCIEU/TwoSampleMR>);  Using the R (version 4.4.0) package ‘coloc’ (<https://github.com/chr1swallace/coloc>);  Using the R (version 4.4.0) package ‘locuscomparer (https://github.com/boxiangliu/locuscomparer). | | |
|  | b) | This study was approved by the Department of Endocrinology, Chengdu University of Traditional Chinese Medicine on May 17, 2024. | | |
|  | **RESULTS** |  | | |
| 10 | **Descriptive data** |  | | |
|  | a) | 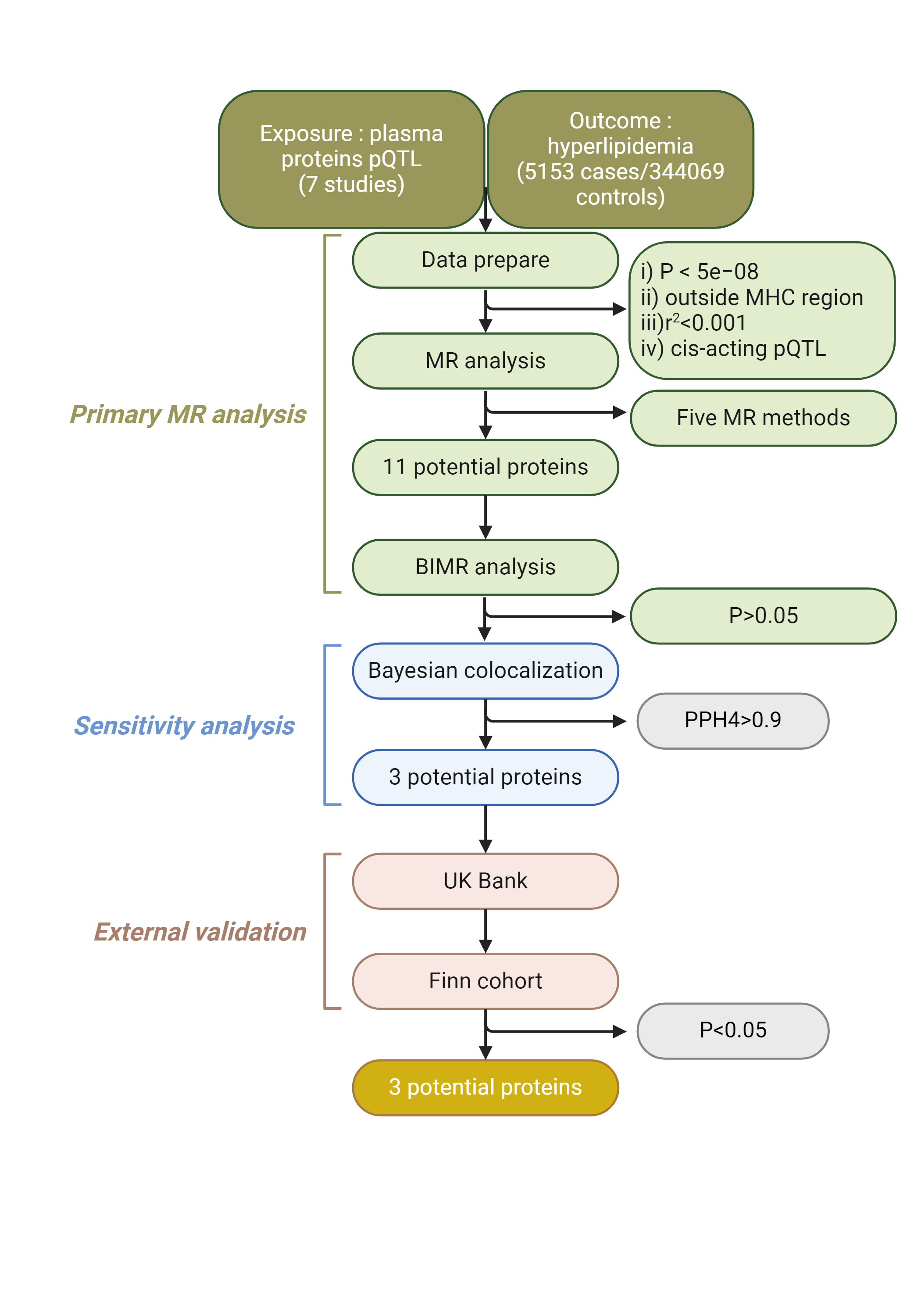 | | |
|  | b) | In the primary MR analysis, pQTL was used as exposure and hyperlipidemia as outcome, and 1839 SNPs were obtained. The F statistics of the harmonized data were all above 10. After BIMR, Bayesian colocalization and Steiger filtering, three potential causal proteins were screened. | | |
|  | c) | i.  The pQTLs we used all had P values ​​lower than 5e-08 and F statistics higher than 10.  ii.  To eliminate heterogeneity and pleiotropy, the pQTLs we used were all cis-pQTLs, which act in transcription and translation. Since most proteins have only one SNP, we performed heterogeneity and pleiotropy analysis on exposure-outcomes with two or more SNPs. The results showed that 18 pQTLs passed the heterogeneity test (Q>0.05) and 2 pQTLs passed the pleiotropy test (P>0.05). | | |
| 11 | **Main results** |  |  |  |
|  | a) | \| **Association between SNP and exposure/outome** \| \| \| \| \| \| \| \| \| --- \| --- \| --- \| --- \| --- \| --- \| --- \| --- \| \| **SNP** \| **exposure** \| **outcome** \| **pval.exposure** \| **pval.outcome** \| **F_statistics** \| **se.exposure** \| **se.outcome** \| \| rs11207970 \| ANGPTL3 \| hyperlipidemia \| 2.13E-96 \| 2.16E-25 \| 401.92 \| 0.01 \| 0.0214982 \| \| rs3135506 \| APOA5 \| hyperlipidemia \| 1.00E-200 \| 8.13E-31 \| 3557.63 \| 0.02 \| 0.0344528 \| \| rs563290 \| APOB \| hyperlipidemia \| 2.00E-13 \| 1.33E-13 \| 69.57 \| 0.01 \| 0.0276929 \| \| rs964184 \| APOC3 \| hyperlipidemia \| 8.39E-27 \| 1.42E-82 \| 107.87 \| 0.02 \| 0.0243911 \| \| rs28399654 \| BCAM \| hyperlipidemia \| 2.09E-232 \| 1.57E-10 \| 994.76 \| 0.02 \| 0.067068 \| \| rs1260326 \| GCKR \| hyperlipidemia \| 7.31E-128 \| 1.02E-43 \| 560.73 \| 0.01 \| 0.0196785 \| \| rs601338 \| KLK1 \| hyperlipidemia \| 0 \| 7.57E-10 \| 22968.89 \| 0.01 \| 0.0200396 \| \| rs2228603 \| NCAN \| hyperlipidemia \| 6.46E-94 \| 7.81E-12 \| 538.71 \| 0.02 \| 0.0425029 \| \| rs11591147 \| PCSK9 \| hyperlipidemia \| 0 \| 3.82E-13 \| 1679.87 \| 0.03 \| 0.103738 \| \| rs1871757 \| TAGLN \| hyperlipidemia \| 2.12E-15 \| 7.73E-10 \| 52.1 \| 0.02 \| 0.0256169 \| \| rs4704826 \| TIMD4 \| hyperlipidemia \| 8.12E-239 \| 3.98E-08 \| 898.42 \| 0.01 \| 0.021141 \| | | |
|  | b) | \| **Primary MR analysis** \| \| \| \| \| \| \| \| \| --- \| --- \| --- \| --- \| --- \| --- \| --- \| --- \| \| **Protein** \| **UniProt ID** \| **SNP** \| **Effect allele** \| **OR (95% CI)** \| **P value** \| **PVE** \| **F statistics** \| \| ANGPTL3 \| Q9Y5C1 \| rs11207970 \| T \| 2.21 (1.91, 2.57) \| 3.88E-25 \| 6.82% \| 401.92 \| \| APOA5 \| Q6Q788 \| rs3135506 \| C \| 1.33 (1.27, 1.39) \| 1.45E-34 \| 34.14% \| 3557.63 \| \| APOB \| P04114 \| rs563290 \| G \| 9.37 (5.12, 17.12) \| 3.58E-13 \| 0.23% \| 69.57 \| \| APOC3 \| P02656 \| rs964184 \| C \| 10.91 (8.69, 13.70) \| 4.41E-94 \| 1.02% \| 107.87 \| \| BCAM \| P50895 \| rs28399654 \| A \| 1.86 (1.52, 2.28) \| 1.65E-09 \| 2.88% \| 994.76 \| \| GCKR \| Q14397 \| rs1260326 \| T \| 0.42 (0.37, 0.47) \| 3.64E-45 \| 8.73% \| 560.73 \| \| KLK1 \| P06870 \| rs601338 \| A \| 1.15 (1.10, 1.20) \| 7.15E-10 \| 18.21% \| 22968.89 \| \| NCAN \| O14594 \| rs2228603 \| T \| 2.34 (1.82, 3.01) \| 4.09E-11 \| 0.76% \| 538.71 \| \| PCSK9 \| Q8NBP7 \| rs11591147 \| T \| 1.81 (1.51, 2.16) \| 6.87E-11 \| 3.90% \| 1679.87 \| \| TAGLN \| Q01995 \| rs1871757 \| A \| 3.44 (2.33, 5.05) \| 3.78E-10 \| 0.39% \| 52.1 \| \| TIMD4 \| Q96H15 \| rs4704826 \| A \| 0.60 (0.50, 0.72) \| 4.43E-08 \| 1.47% \| 898.42 \| | | |
|  | c) | BIMR  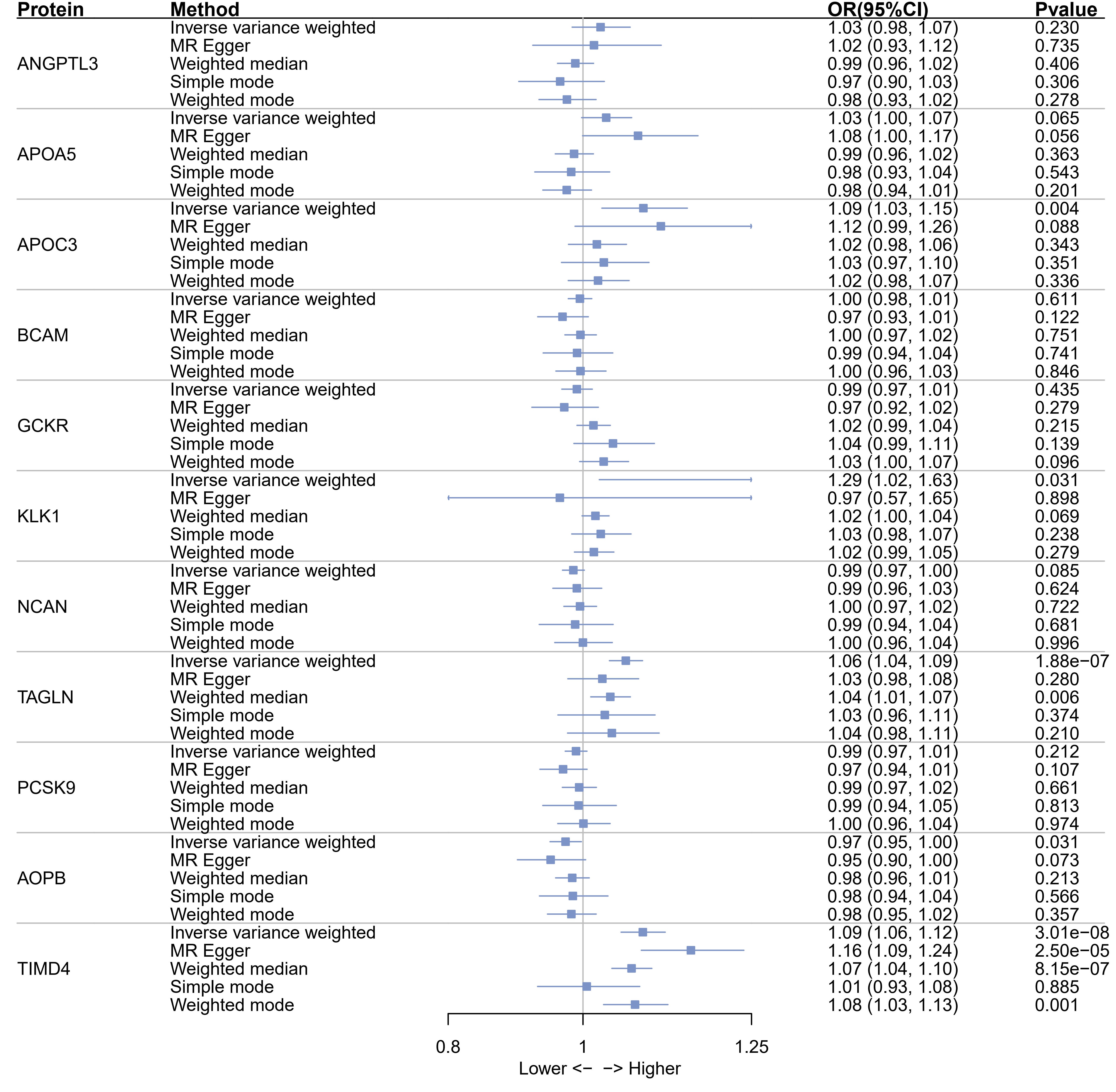 | | |

|  | d) | Bayesian colocalization  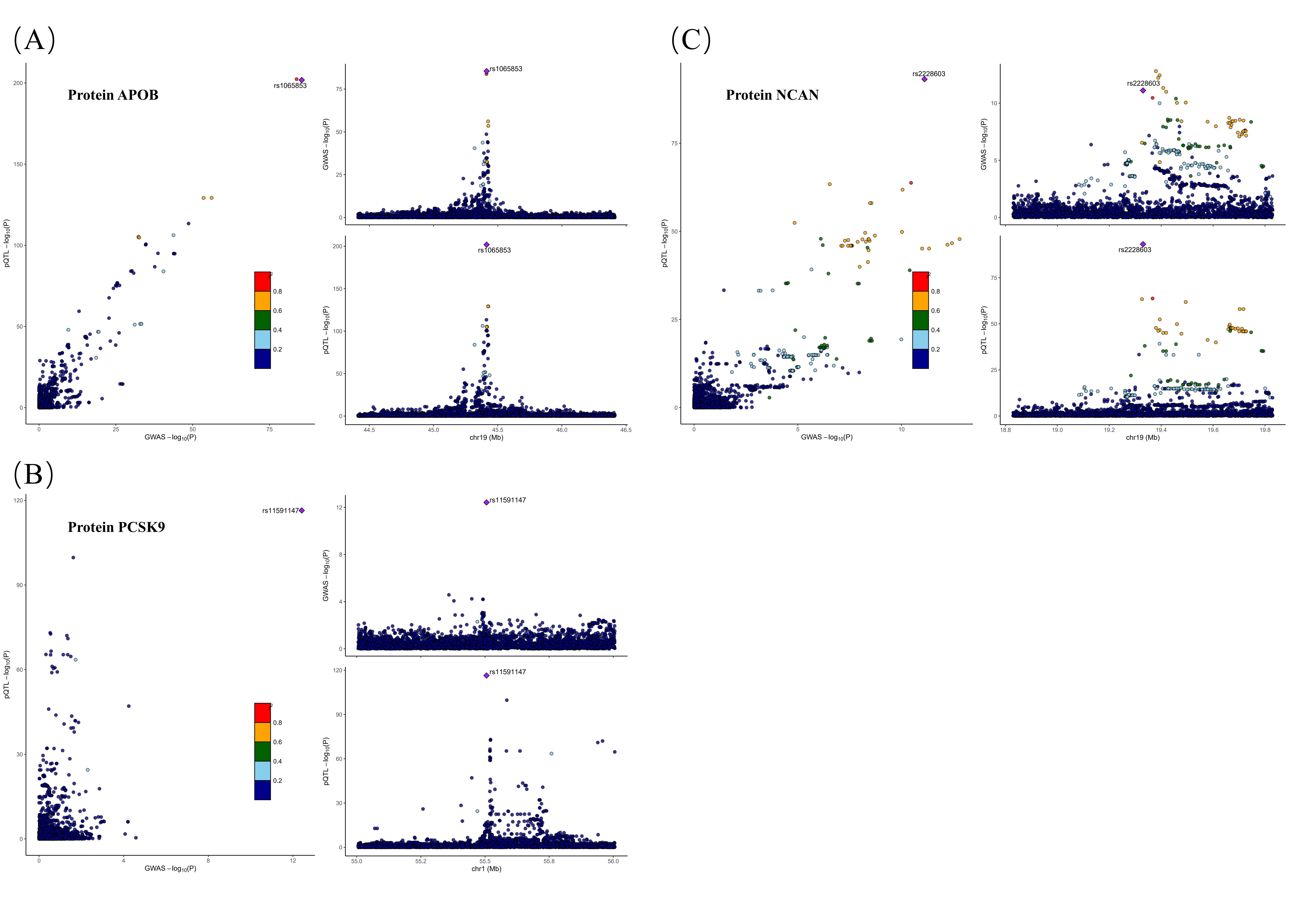 | | |
| --- | --- | --- | --- | --- |
| 12 | **Assessment of assumptions** | PVE and F statistics are provided in the primary MR | | |
| 13 | **Sensitivity analyses and additional analyses** | Sensitivity analysis and bidirectional MR are provided in the above results | | |
|  | **DISCUSSION** |  |  |  |
| 14 | **Key results** | After MR, BIMR, sensitivity analysis, heterogeneity analysis, Bayesian colocalization, Steiger filtering and external validation, the results showed that circulating plasma APOB, PCSK9 and NCAN were potential causal proteins of hyperlipidemia. |  | ‘According to the Bonferroni significance test (P < 5e-08), MR analysis demonstrated a causal relationship between 11 proteins and hyperlipidemia (Table 1 and Figure 2C), including angiopoietin-related protein 3 (ANGPTL3), apolipoprotein A-V (APOA5), apolipoprotein C-III (APOC3), apolipoprotein B (APOB)-100, basal cell adhesion molecule (BCAM), glucokinase regulatory protein (GCKR), kallikrein-1 (KLK1), neurocan core protein (NCAN), protein convertase subtilisin/kexin type 9 (PCSK9), transgelin (TAGLN), and T-cell immunoglobulin and mucin domain-containing protein 4 (TIMD4). Specifically, increased levels of GCKR (odds ratio [OR] = 0.42, 95% confidence interval [CI], 0.37–0.47; P = 3.64e-45) and TIMD4 (OR = 0.60, 95% CI, 0.50–0.72; P = 4.43e-08) decreased the risk of hyperlipidemia. In contrast, incensed levels of ANGPTL3 (OR = 2.21, 95% CI, 1.91–2.57; P = 3.88e-25), APOA5 (OR = 1.33, 95% CI, 1.27–1.39; P = 1.45e-34), APOB (OR = 9.37, 95% CI, 5.12–17.12; P = 3.58e-13), APOC3 (OR = 10.91, 95% CI, 8.69–13.70; P = 4.41e-94), BCAM (OR = 1.86, 95% CI, 1.52–2.28; P = 1.65e-09), KLK1 (OR = 1.15, 95% CI, 1.10–1.20; P = 7.15e-10), NCAN (OR = 2.34, 95% CI, 1.82–3.01; P = 4.09e-11), TAGLN (OR = 3.44, 95% CI, 2.33–5.05; P = 3.78e-10), and PCSK9 (OR = 1.81, 95% CI, 1.51–2.16; P = 6.87e-11) indicated higher risk of hyperlipidemia.’  ‘Our initial findings revealed that of the eleven causal proteins, nine possessed the potential to serve as drug targets for treating hyperlipidemia. Following the screening of the preliminary analysis results for bidirectional causality, any MR analysis meeting a P-value of <0.05 was considered to have reverse causality (Figure 3A). Four potential therapeutic drug targets were identified: APOB, PCSK9, BCAM, and NCAN. Steiger filtering ensures directionality, as listed in Table 2. Bayesian colocalization of three of the four proteins indicated a common variant for hyperlipidemia (Supplementary Figure 1), specifically, APOB (PPH4) = 0.997, NCAN (PPH4 = 0.932), and PCSK9 ([PPH4 = 1], Figure 3B). Additionally, we performed a co-localization analysis of each protein, as shown in Supplementary Figure 2. Notably, PCSK9 shares the same variant as APOB (rs11541192).’ |
| 15 | **Limitations** | The limitations of our study should be acknowledged, including the potential bias arising from the pQTL data sourced from seven different studies. The circulating protein GWAS data were all based on aptamers known for their high specificity and stability in binding to target molecules. Cis-pQTL were chosen and only have one SNP was considered, while some trans-pQTL were not assessed, which limits the applicability of alternative MR, multiplicity testing, and heterogeneity detection. However, the SNPs utilized in our research have been established as strong instrumental variables with F-statistic values exceeding 10, which lends credibility to our statistical analysis. Third, the data samples employed in our study were derived from European populations, making it challenging to generalize our findings to other populations. Further investigation using more individual data is needed for the effective clinical translation of naringenin in treatment of hyperlipidemia. We also confirmed a causal relationship between APOB, PCSK9, and NCAN proteins and hyperlipidemia in the external dataset. Further research in non-European populations is required. |  | ‘The limitations of our study should be acknowledged, including the potential bias arising from the pQTL data sourced from seven different studies.’ |
| 16 | **Interpretation** |  |  |  |
|  |  | Human proteins are major therapeutic targets of drugs. To identify whether the naringenin target were causal proteins of hyperlipidemia. We adopted a combination of MR and co-localization analyses to interpret previous GWAS studies on hyperlipidemia. The "causality" identified by MR may be horizontally pleiotropic or contain reverse causality and genetic confounding. Therefore, proteins with reverse causality were further excluded by bidirectional Mendelian randomization (BIMR), and results of Steiger filtering supported our primary findings. We eliminated the bias of horizontal pleiotropy as much as possible by using only cis-pQTL as instrumental variables. In addition, by applying a Bayesian co-localization threshold of 0.9 for posterior probability, the bias resulting from genetic confounders was successfully mitigated. The three proteins identified by co-localization (PCSK9, APOB, and NCAN) may share the same variant.  To the best of our knowledge, we are the first to reveal causal relationship between naringenin and hyperlipidemia by utilizing the compound-target-disease network and MR. Here, we report three potential drug-targeting proteins for hyperlipidemia: PCSK9, APOB, and NCAN. Among these proteins, the association between APOB and hyperlipidemia was also validated using external datasets, which make the results more reliable. Consistent with our findings, previous studies have shown that SNPs of APOB are associated with hyperlipidemia in Chinese and Finnish populations. Human APOB is the major protein component of LDL (apolipoprotein B-100), chylomicron (apolipoprotein B-48) and VLDL (apolipoprotein B-100), and plays a crucial role in maintaining healthy cholesterol levels. Plasma APOB is equal to the total number of APOB48, APOB100 particles, and chylomicron. Lipid particles that contribute to hyperlipidemia are typically determined by the number of APOB present in blood vessels. LDL particles, which have higher cholesterol content, are more likely to deposit cholesterol and increase the risk of cardiovascular disease. In our study, we observed a significant interaction between APOB and LDLR, which led us to propose that the effects of naringenin may be due to the co-regulation of these two proteins.  The results of the combined MR analyses indicate that PCSK9 is a promising therapeutic target, as its inhibitor, alirocumab, has been approved by FDA. Additionally, the feasibility of targeting PCSK9 was demonstrated using the newly approved hypercholesterolemic nucleic acid-lowering drug, inclisiran. These findings support our hypothesis that naringenin modulates OS and lipid metabolism during the treatment of hyperlipidemia, as shown by the results of the MR analysis of PCSK9 with APOB. It is also worth noting that the interaction between NCAN and the environment has been linked to hyperlipidemia, and these studies further support the results of our research. |  | ‘Human proteins are major therapeutic targets of drugs.’  ‘To the best of our knowledge, this is the first study to reveal a causal relationship between naringenin and hyperlipidemia using a compound-target-disease network and MR. Herein, we report three potential drug-targeting proteins for hyperlipidemia: PCSK9, APOB, and NCAN (Table 2).’ |
|  |  |  |  |  |
|  |  |  |  |  |
| 17 | **Generalizability** | The data samples employed in our study were derived from European populations, making it challenging to generalize our findings to other populations. Further investigation using more individual data is needed for the effective clinical translation of naringenin in treatment of hyperlipidemia. |  | ‘the data samples employed in our study were derived from European populations, making it challenging to generalize our findings to other populations.’ |
|  | **OTHER INFORMATION** |  |  |  |
| 18 | **Funding** | This work is supported by “Xinglin Scholar” of Chengdu University of Traditional Chinese Medicine (QJRC2023010, YYZX2022016); Sichuan Traditional Chinese Medicine Culture Collaborative Development Research Center (2023XT17). |  |  |
| 19 | **Data and data sharing** | The following sources provide access to genome-wide summary level statistics for cis-pQTL: the original study, the IEN OpenGWAS, and the UK Biobank. The IEN OpenGWAS summary statistics can be accessed at (https://gwas.mrcieu.ac.uk/), while the UK Biobank's GWAS summary statistics can be obtained from (https://www.leelabsg.org/). Additionally, the FinnGen (version R10) dataset can be accessed at (<https://www.finngen.fi/en/access_results>).^1-9^  All R codes can be obtained from the author Jian Gao ([masogotsucked@gmail.com](mailto:masogotsucked@gmail.com)).  QuanYu Du conceived of the study, Jian Gao participated in the design and coordination, Huanyu Jiang, Wenjia Xian, Ganggang Li and Linjie Yuan completed the data analysis, Jian Gao completed the manuscript drafting, and Yutong Zou, Yuwei Zhang and Ruijun Zhou completed the picture drawing. QuanYu Du and Xianhua Zhou read and edited the final version of the manuscript. All authors contributed to the revision and reading of the manuscript and approved the version as submitted. |  |  |
| 20 | **Conflicts of Interest** | The authors declare that the research was conducted in the absence of any commercial or financial relationships that could be construed as potential conflicts of interest. |  |  |

This checklist is copyrighted by the Equator Network under the Creative Commons Attribution 3.0 Unported (CC BY 3.0) license.

**References:**

1. Sun BB, Maranville JC, Peters JE et al. Genomic atlas of the human plasma proteome. *Nature*. 2018;558(7708):73-79

2. Ferkingstad E, Sulem P, Atlason BA et al. Large-scale integration of the plasma proteome with genetics and disease. *Nat Genet*. 2021;53(12):1712-1721

3. Pietzner M, Wheeler E, Carrasco-Zanini J et al. Mapping the proteo-genomic convergence of human diseases. *Science*. 2021;374(6569):eabj1541

4. Sun BB, Chiou J, Traylor M et al. Plasma proteomic associations with genetics and health in the UK Biobank. *Nature*. 2023;622(7982):329-338

5. Suhre K, Arnold M, Bhagwat AM et al. Erratum: Connecting genetic risk to disease end points through the human blood plasma proteome. *Nat Commun*. 2017;8:15345

6. Yao C, Chen G, Song C et al. Genome-wide mapping of plasma protein QTLs identifies putatively causal genes and pathways for cardiovascular disease. *Nat Commun*. 2018;9(1):3268

7. Folkersen L, Fauman E, Sabater-Lleal M et al. Mapping of 79 loci for 83 plasma protein biomarkers in cardiovascular disease. *PLoS Genet*. 2017;13(4):e1006706

8. Sudlow C, Gallacher J, Allen N et al. UK biobank: an open access resource for identifying the causes of a wide range of complex diseases of middle and old age. *PLoS Med*. 2015;12(3):e1001779

9. Kurki MI, Karjalainen J, Palta P et al. FinnGen provides genetic insights from a well-phenotyped isolated population. *Nature*. 2023;613(7944):508-518
