# Supplementary material for: Naringenin modulates oxidative stress and lipid metabolism: Insights from network pharmacology, mendelian randomization, and molecular docking
Source: Front Pharmacol. 2024 Oct 15;15:1448308. doi: 10.3389/fphar.2024.1448308 (PMC11518751; doi:10.3389/fphar.2024.1448308)
Supplement: Supplementary file 7 [file DataSheet1.docx]

Supplementary Material

**Supplementary Table 1 Targets of Naringenin in TCMSP/HERB/BATMAN and intersection with phenotypes in GeneCards dataset**

**Supplementary Table 2 Targets with "oxidative stress" phenotype relevance score greater than 20 in GeneCards dataset**

**Supplementary Table 3 Targets with "lipid metabolism" phenotype relevance score greater than 20 in GeneCards dataset**

**Supplementary Table 4 Genetic instruments of plasma proteins for MR analysis**

**Supplementary Table 5 Genetic instruments of hyperlipidemia for bidirectional MR**

**Supplementary Table 6 Bayesian colocalizations three of potential causal proteins and hyperlipidemia**

**Supplementary Table 7 Protein-protein interaction network among the naringenin potential targets**

**Supplementary Table 8 Protein-disease network among the naringenin potential targets**

**Supplementary Table 9 Protein-protein causal relationship among PCSK9 and APOB**

**Supplementary Table 10 MR analysis of three potential causal proteins for external validation**

**All Supplementary Tables are provided in an excel file (“Supplementary Table 1.xlsx”).**

**Supplementary Fig. 1 Bayesian colocalization analyses of three potential causal proteins and hyperlipidemia**

**
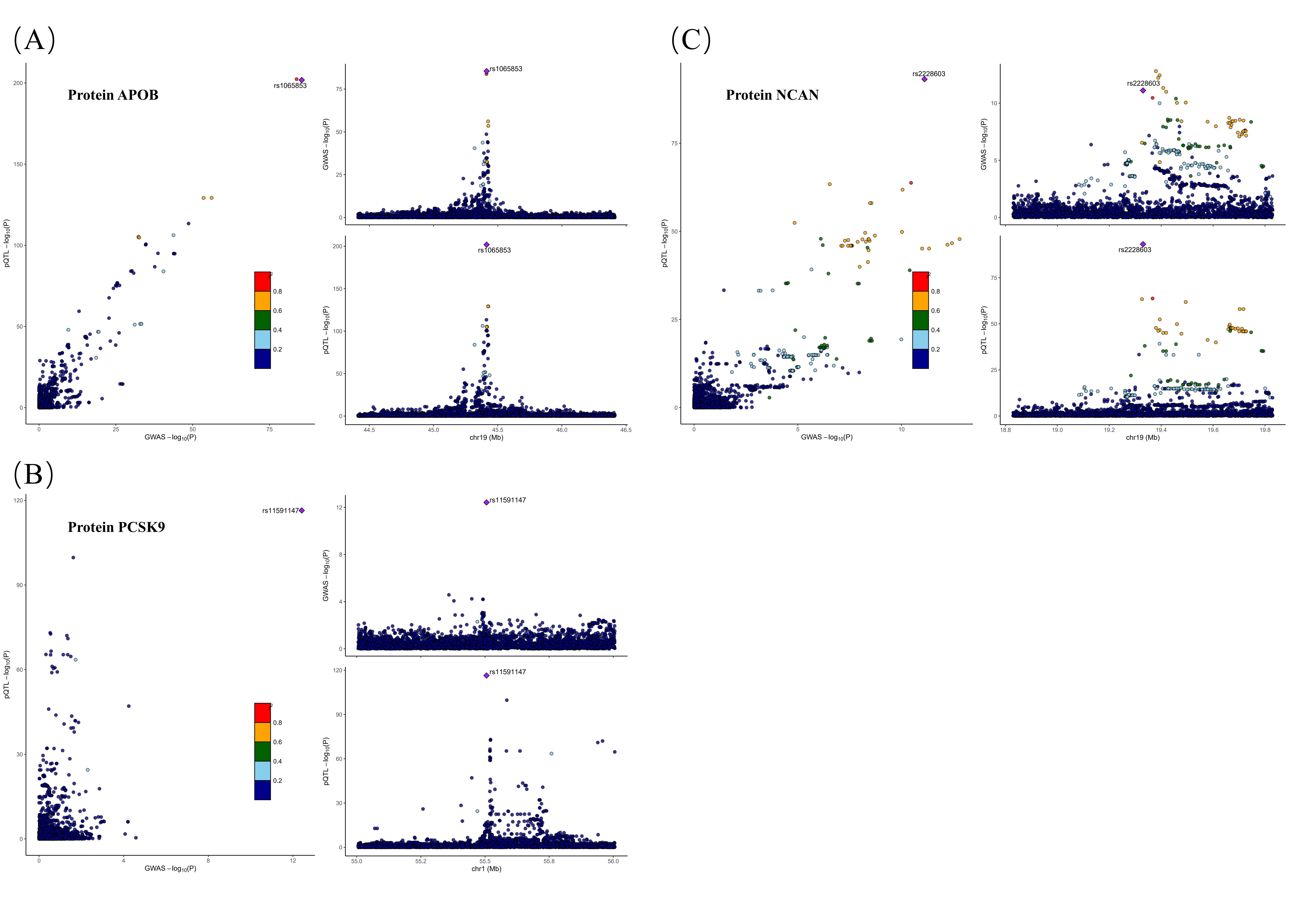
**

Colocalization analyses of plasma proteins for APOB (A), PCSK9 (B) and NCAN (C), respectively. Diamond purple points represented the SNP that with the minimal sum of P value in corresponded protein GWAS and hyperlipidemia GWAS.

**Supplementary Fig. 2 Protein-protein causal relationship among three potential causal proteins**

**
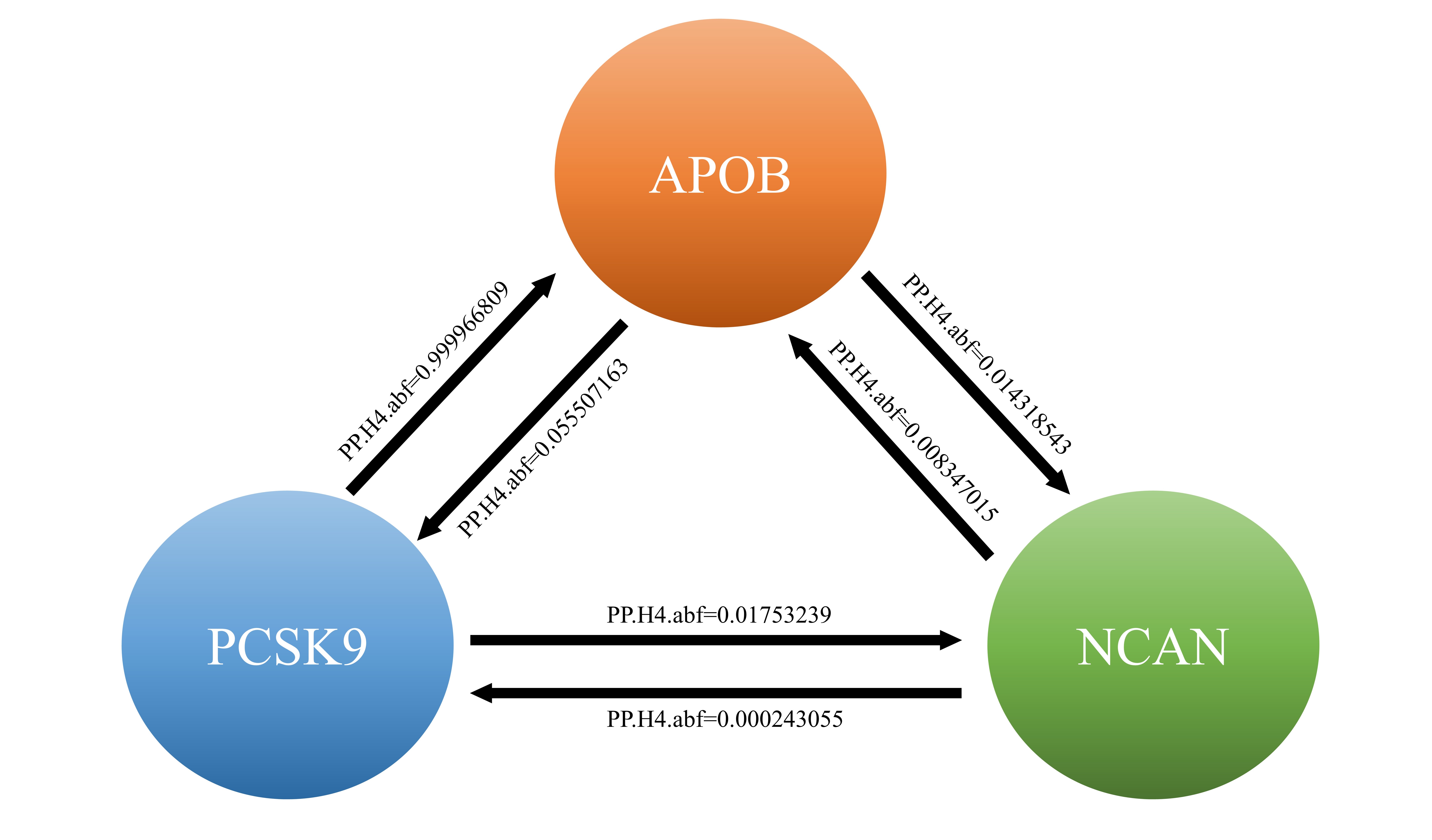
**

Colocalization analysis of among APOB, PCSK9 and NCAN, respectively.

PP.H4.abf = coloc.abf-posterior probability of hypothesis 4

**Supplementary Fig. 3 Targets of Naringenin in TCMSP/HERB/BATMAN and intersection with phenotypes in GeneCards dataset**

**
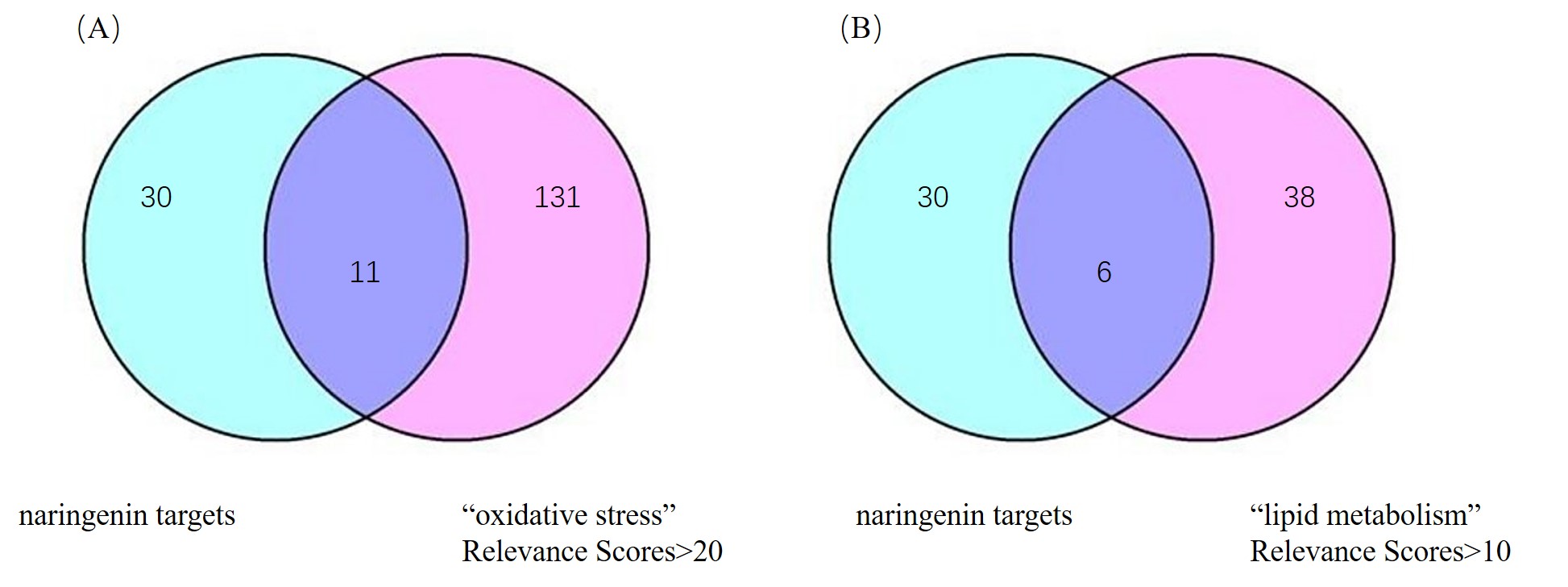
**

1. Naringenin potential targets with "oxidative stress" phenotype, (B) Naringenin potential targets with "lipid metabolism" phenotype.

**Supplementary Fig. 4 Protein-protein interaction network among the three potential causal proteins**

**
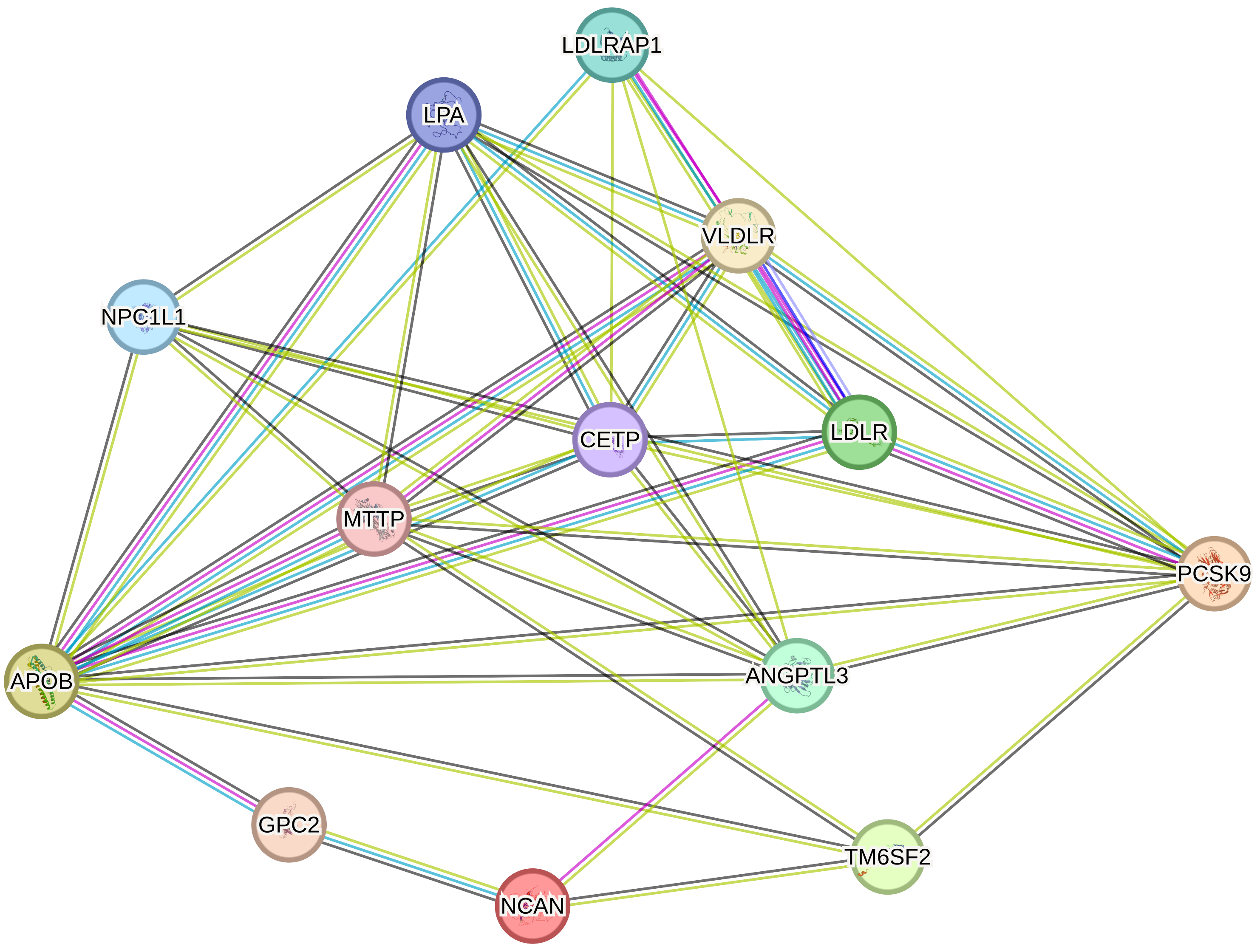
**

Lines represent protein associations. Black lines represent co-expression. Blue lines represent gene co-occurrence. Purple lines represent experimentally determined. Light blue lines represent from curated databases. Dark green lines represent gene neighborhood. Cyan represents protein homology. Light green represents textmining.
